# A focused review of statistical practices for relating radiation dose-volume exposure and toxicity

**DOI:** 10.1186/s13014-023-02220-9

**Published:** 2023-03-24

**Authors:** Andrew M. McDonald, Craig S. Schneider, John M. Stahl, Robert A. Oster, Richard A. Popple, Charles S. Mayo

**Affiliations:** 1 Department of Radiation Oncology, Hazelrig-Salter Radiation Oncology Center, University of Alabama at Birmingham, Birmingham, AL USA; 2 Institute for Cancer Outcomes and Survivorship, University of Alabama at Birmingham, Birmingham, AL USA; 3 Department of Medicine, Division of Preventive Medicine, University of Alabama at Birmingham, Birmingham, AL USA; 4 Department of Radiation Oncology, University of Michigan, Ann Arbor, MI USA

## Abstract

**Purpose:**

Relating dose-volume histogram (DVH) information to patient outcomes is critical for outcomes research in radiation oncology, but this is statistically challenging. We performed this focused review of DVH toxicity studies to characterize current statistical approaches and determine the need for updated reporting recommendations.

**Methods and materials:**

We performed a focused MEDLINE search to identify studies published in 5 radiation oncology specialty journals that associated dosimetry with toxicity outcomes in humans receiving radiotherapy between 2015 and 2021. Elements abstracted from each manuscript included the study outcome, organs-at-risk (OARs) considered, DVH parameters analyzed, summary of the analytic approach, use of multivariable statistics, goodness-of-fit reporting, completeness of model reporting, assessment of multicollinearity, adjustment for multiple comparisons, and methods for dichotomizing variables. Each study was also assessed for sufficient reporting to allow for replication of results.

**Results:**

The MEDLINE search returned 2,300 studies for review and 325 met the inclusion criteria for the analysis. DVH variables were dichotomized using cut points in 154 (47.4%) studies. Logistic regression (55.4% of studies) was the most common statistical method used to relate DVH to toxicity outcomes, followed by Cox regression (20.6%) and linear regression (12.0%). Multivariable statistical tests were performed in 226 (69.5%) studies; of these, the possibility of multicollinearity was addressed in 47.8% and model goodness-of-fit were reported in 32.6%. The threshold for statistical significance was adjusted to account for multiple comparisons in 41 of 196 (17.1%) studies that included multiple statistical comparisons. Twenty-eight (8.6%) studies were classified as missing details necessary to reproduce the study results.

**Conclusions:**

Current practices of statistical reporting in DVH outcomes suggest that studies may be vulnerable to threats against internal and external validity. Recommendations for reporting are provided herein to guard against such threats and to promote cohesiveness among radiation oncology outcomes researchers.

**Supplementary Information:**

The online version contains supplementary material available at 10.1186/s13014-023-02220-9.

## Introduction

Effective implementation of continually advancing radiotherapy delivery techniques requires evidence-based treatment planning goals to reduce patient complications. The first step to deriving these treatment planning goals is to establish an understanding of the relationship between radiation exposure and clinical outcomes. The most common method of quantifying radiation exposure to an organ at risk (OAR) is the cumulative dose volume histogram (DVH). When characteristics of the DVH can be related to patient outcomes, specific DVH parameter goals can be used during treatment plan creation to reduce the risk of complications.

Conventional statistical methods operating on a set of values are not implicitly matched to the set of paired dose and volume values characteristic of a DVH curve. A few authors have introduced approaches to regularize the analysis of sets of dose-volume pairs along with outcomes, such as the Atlas of Complication Incidence by Jackson et al., [1] but the most common general strategy to overcome this problem has instead been to focus one at a time on a component of the pair (e.g. mean dose, maximum dose, or volume receiving a particular dose) in order to facilitate the use of statistical tests that are more familiar in biomedical research. However, within this general framework, specific statistical methods employed can be inconsistent between studies, potentially producing inconsistent findings and muddling future meta-analyses. This inconsistency can lead to confusion for clinicians attempting to incorporate findings from these studies into practice.

Incomplete and inconsistent reporting of statistical methods used in DVH studies has been recognized as an important problem. In the collection of recommendations from Quantitative Analysis of Normal Tissue Effects and Complications (QUANTEC), authors for each organ analysis paper identified specific DVH metric values that, if routinely collected and presented in published analysis of toxicities, would support inter-comparison and benchmarking of results among studies. Issues in overall reporting standards and statistical requirements and recommendations from QUANTEC to address these problems were detailed by Jackson et al. [2]

Greater focus has also been placed on statistical methodology and reporting throughout healthcare research as reflected by the work of the Enhancing the Quality and Transparency of Health Research (EQUATOR) network, and more specifically the development of the Transparent Reporting of a multivariable prediction model for Individual Prognosis Or Diagnosis (TRIPOD) and Strengthening the Reporting of Observational studies in Epidemiology (STROBE) statements [3–5]. However, there has been a lack of published studies characterizing statistical practices used in studies measuring associations of DVH metrics with toxicity. The effect that research quality initiatives have had on statistical methodology and comprehensive reporting in this field is also underreported. We therefore performed this focused review DVH toxicity studies to characterize current statistical approaches and reporting practices.

## Methods and materials

### Study design and search strategy

We performed this focus review by following principles of the PRISMA statement [6]. Study selection and data abstraction were guided by a written protocol. The protocol was designed to assess general review questions about statistical methods used to relate DVHs with clinical outcomes:

- How are DVH parameters chosen for analysis?
- What are the most common statistical approaches used to assess the relationship between DVHs and clinical outcomes?
- What is the relative frequency of each statistical approach?
- For studies using multivariable statistical tests containing multiple DVH parameters, how often is multicollinearity between variables is assessed?
- For studies performing multiple analyses, how often are adjustments for multiple comparisons made?
- What is the frequency of reporting goodness-of-fit and other model statistics?
- If threshold cut points were used for analysis, were they arrived at statistically using the study data set or were they drawn from prior publications?

### Study inclusion and search strategy

Studies published in the 7-year interval of 2015 to 2021 were eligible for inclusion if the abstract reported statistical tests assessing the effect of DVH parameters on toxicity outcomes in humans. Studies that exclusively parameterized NTCP modeling equations and did not perform conventional statistical tests were excluded. Studies were identified by searching MEDLINE using PubMed with the search string [Dose Volume Histogram] OR [DVH] OR [Normal Tissue Complications] OR [Organ At Risk] OR [Dosimetry Analysis] OR [Dosimetric Analysis] OR [Toxicity] OR [Morbidity]. The search results were filtered to include only articles from 5 journals with a primary radiation oncology readership: *Acta Oncologica, The International Journal of Radiation Oncology · Biology · Physics, Radiotherapy and Oncology, Radiation Oncology,* and *Practical Radiation Oncology.*

### Selection of studies and data collection

Search results were imported into Covidence software and the abstract of each study identified by the search was reviewed for content by one reviewer (AM). Studies whose abstracts indicated that analyses relating dosimetric information to patient outcomes had been performed were selected for further review of the manuscript and any available Additional file 1. Manuscripts were reviewed by 2 reviewers (AM and CS). The elements abstracted from each manuscript that met the inclusion criteria were: PMID, year of publication, journal, type of radiation technique utilized, toxicity outcome, data source, generation of NTCP, general analytic approach used, OARs analyzed, DVH parameters analyzed, justification for choice of DVH parameters, use of multivariable analysis, selection of variables for multivariable analysis, reporting of goodness-of-fit statistics for models, reporting of entire models, assessment for multicollinearity, adjustments for multiple comparisons, inclusion of non-dosimetric variables, and techniques for dichotomizing variables.

Each study was also assessed for sufficient reporting to allow for replication of results. Reviewers familiar with the STROBE and TRIPOD statements [3, 5] were prompted by the statement: “Assuming access to the study data and collaboration with an expert biostatistician, is enough detail provided to replicate the study results?” If any reviewer classified a manuscript as having insufficient reporting, then it was independently reviewed by 3 other reviewers, including an expert biostatistician. Only studies where all 3 reviewers (AM, CS, and JS) and the biostatistician (RO) unanimously agreed were classified in this review as having insufficient reporting.

## Results

### Search results and overview of included studies

**Fig. 1:**
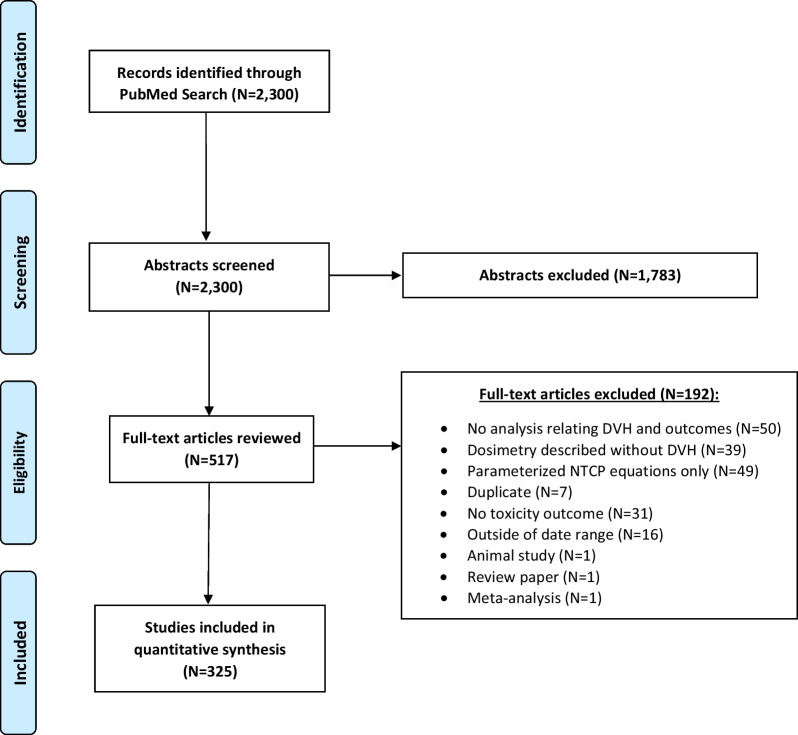
Flow diagram of study inclusion

The PubMed query returned a total of 2,300 articles. After abstract review, 1,783 studies were excluded and 517 studies were selected for manuscript review. An additional 192 studies were excluded after reviewing the manuscript, resulting in a total of 325 studies included. The flow diagram of study inclusion is presented as Fig. 1. Complete bibliographic information of all studies included in this review is provided in the Additional file 2. A descriptive summary of the included studies is presented as Table 1. Outcomes data were most commonly from retrospective cohorts (68.9%), followed by clinical trials (14.8%) and prospective cohorts (12.0%). Most studies focused exclusively on the dosimetry of external beam radiation. The median analytic sample size was 119, ranging from 12 to 24,214. The sample size for dosimetric analysis was typically equal to the number of patients, but a minority of studies treated each OAR as a case (e.g. each kidney analyzed separately).

**Table 1:** Summary of general study and publication characteristics

Characteristic	N(%) of 325
*Journal*	
Int J Radiat Oncol Biol Phys	103 (31.7)
Radiother Oncol	103 (31.7)
Pract Radiat Oncol	36 (11.1)
Radiat Oncol	55 (16.9)
Acta Oncol	28 (8.6)
*Year of Publication*	
2015	46 (14.2)
2016	49 (15.1)
2017	59 (18.2)
2018	43 (13.2)
2019	55 (16.9)
2020	31 (9.5)
2021	42 (12.9)
*Data Source*	
Clinical trial	48 (14.8)
Prospective cohort	39 (12.0)
Retrospective cohort	224 (68.9)
Case control	8 (2.4)
Other	6 (1.8)
*Sample Size*	
< 100	124 (38.2)
100–499	157 (48.3)
500–999	29 (8.9)
≥ 1,000	15 (4.6)
*Radiation Modality*	
External Beam	300 (92.3)
Brachytherapy	15 (4.6)
Both	10 (3.1)
*Study Outcome*^*1*^	
Pulmonary morbidity	41 (12.6)
Impaired liver function	10 (3.1)
Lower GI morbidity	46 (14.2)
Urinary morbidity	33 (10.2)
Brain necrosis	17 (5.2)
Hematologic morbidity	24 (7.4)
Cardiac morbidity	22 (6.8)
Chest wall pain	9 (2.8)
Esophagitis	14 (4.3)
Xerostomia	11 (3.4)
Swallowing function	13 (4.0)
Non-cancer death	13 (4.0)
Other	120 (36.9)

^1^Total number of study outcomes exceeds 325 due to *N* = 40 studies assessing 2+ outcome variables and *N* = 10 studies assessing 3+ outcome variables

### Dose-volume parameters and non-dosimetric independent variables

The OARs and OAR sub-regions assessed were explicitly defined in all studies. Forty-six (14.2%) studies provided specific justification for the choice of DVH parameters assessed, with the most common rationale that parameters had been included in prior research studies (11.7% of studies). A single structure was the subject of investigation in 194 (59.7%) studies and 2 structures in 55 (16.9%). More than 5 structures were assessed in 26 (8.0%) studies; however, in most cases this was accounted for by sub-segmenting a single OAR into separate regions of interest.

The DVH parameters chosen for analysis were explicitly stated in all but 3 studies. The median number of total DVH parameters analyzed was 8 and ranged from 1 to > 1,000. The median number of DVH parameters considered per structure was 6 and ranged from 1 to approximately 80. For some studies, the exact number of DVH parameters analyzed could not be exactly calculated even though the definitions were explicit, such as when parameters were extracted incrementally across a DVH but the maximum dose extracted was not provided. In addition to dosimetric parameters, non-dosimetric variables were considered in 242 (74.5%) studies.

### Study outcomes

Most studies assessed only 1 toxicity domain, but 40 (12.3%) studies assessed 2 outcome domains and 10 (3.1%) assessed 3 toxicity domains. For the purposes of dosimetric analysis, outcomes were considered as binary variables in 286 (88.0%) studies, continuous or ordinal variables in 28 (8.6%) studies, and a combination of types in the remaining studies.

### General analytic approaches and techniques

A summary of the analytic techniques used to associate DVH information with patient toxicity outcomes is summarized in Table 2. We observed that 102 (31.3%) studies included a non-directional test comparing DVH parameters between the cohort of patients who experienced toxicity and the cohort who did not, typically with a test of means such as T-test. Results from these comparisons were then often used to identify the DVH parameters to be investigated further with other statistical tests. Actuarial statistical tests that considered the timing of the toxicity endpoint were utilized in 95 (29.2%) studies (e.g. Cox regression or Kaplan–Meier), and the remaining studies only used tests that were agnostic to the timing of the endpoint (e.g. logistic or linear regression). Logistic regression was the overall most common statistical test used to relate DVH to toxicity outcomes and was used in 180 (55.4%) studies. The other 2 most common tests used were Cox regression in 67 (20.6%) studies and linear regression in 39 (12.0%) studies.

**Table 2:** Summary of statistical methods

Characteristic	N (%) of 325
*Analytic approach*^*1*^	
Non-directional test of means	102 (31.3)
Logistic regression	180 (55.4)
Linear regression	39 (12.0)
Cox proportional hazards	67 (20.6)
Other	75 (23.1)
*No. of OARs assessed*	
1	194 (59.7)
2	55 (16.9)
≥ 3	76 (23.3)
*No. of DVH parameters assessed*	
1–5	109 (33.5)
6–20	141 (43.4)
21–100	54 (16.6)
> 100	18 (5.5)
Not explicitly stated	3 (0.9)
*Method of selecting DVH parameters*	
Prior research	28 (11.7)
Institutional treatment protocol	18 (5.5)
Not stated	279 (85.8)
*Multivariable statistics*	
Yes	226 (69.5)
No	99 (30.5)
*Multiple comparisons performed*	
Yes	258 (79.4)
No	67 (20.6)
*Correction for multiple comparisons*^*2*^	
None	214 (82.9)
Bonferroni	15 (5.8)
Bootstrapping	12 (3.7)
Benjamini-Hochberg	5 (1.5)
Cross validation	3 (0.9)
Other	9 (2.8)
*Cut points used*	
Yes	154 (47.4)
No	171 (52.6)
*Method of determining cut points*^*3*^	
Prior research or protocol	8 (5.2)
Set to mean, median, or quartile values	17 (11.0)
Maximize discriminant value from ROC curve	63 (40.9)
Recursive partitioning	9 (5.8)
Not stated	23 (14.9)
Other	37 (24.0)
*Non-dosimetric variables included*	
Yes	242 (74.5)
No	83 (25.5)

^1^Total number sums to greater than 100% due to some studies using multiple types of analyses

^2^Denominator adjusted to reflect the 258 studies with multiple comparisons

^3^Denominator adjusted to reflect the 154 studies using cut points and numbers do not sum to 154 due to 3 studies that utilized both recursive partitioning and ROC methods to establish cut points

Statistical analysis included multiple comparisons in 258 (79.4%) studies. The threshold for statistical significance was adjusted to account for multiple comparisons in 44 (17.1%) studies, most commonly with Bonferroni adjustments in 15 (5.8%) studies and bootstrap internal validation in 12 (3.7%) studies.

DVH variables were dichotomized using cut points in 154 (47.4%) studies, and the method by which cut points were selected could be determined in 131 studies. Cut points were pulled from prior research in 8 studies and determined based on the distribution of DVH parameters in 17 studies (e.g. cut point at the median value). The remaining studies selected cut points through the use of statistical techniques that maximized the discriminant ability of the cut point, the most common of which was using a dichotomization metric derived from receiver operator characteristic (ROC) curves (e.g. Youden’s index, concordance probability) in 63 studies.

### Multivariable statistics

Multivariable statistical tests that included one or more DVH parameter as a predictor variable were performed in 226 (69.5%) studies, and of these studies, the method used to construct multivariable models and choose the final model could be determined for 184 (Table 3). Forward selection was the most common method in 114 studies, followed by backward elimination in 32 studies, and the remaining studies used a hybrid or alternative strategy. Measures of model fit were reported by 105 studies.

**Table 3:** Description of multivariable statistical techniques used in 226 studies

Characteristic	*N* (%) of 226
*Variable selection strategy*	
Forward selection	114 (50.4)
Backward elimination	32 (14.2)
Bidirectional	9 (4.0)
Entry method	16 (7.1)
Other	13 (5.8)
Not stated	42 (18.6)
*Method of multicollinearity assessment*	
Pearson and/or Spearman correlation	45 (19.9)
Assumption of multicollinearity	36 (15.9)
Other	27 (11.9)
Not addressed	118 (52.2)

The possibility of multicollinearity between predictor variables was specifically addressed in 108 of 226 (47.8%) studies reporting multivariable statistics. Statistical tests to assess multicollinearity were reported in 72 studies, most commonly with either Pearson or Spearman correlation, and the remaining studies assumed collinearity between DVH parameters and only allowed a single DVH parameter within each multivariable model.

### Results reporting and assessment of replicability

Incomplete reporting of statistical analyses were identified in 88 (27.1%) studies. Examples of incomplete results reporting included omission of non-significant variables from univariate analyses tables, reporting only selected variables within a multivariable model, or only reporting selected models when multiple models were performed. In most instances the omitted results were noted by the reviewers but did not alter the overall impression or interpretation of the study; however, key results needed could not be identified in 11 (3.4%) studies which led to reviewers classifying these studies as not able to be replicated based on the information provided. An additional 17 (5.2%) studies were classified as not able to be replicated on the basis of important details about the statistical analysis not being provided.

## Discussion

Determining how radiation exposure to OARs influences the probability of toxicity after radiation treatment is a critical step for improving future treatment planning but relating dosimetry information to patient outcomes is methodologically challenging. A strong analytic plan should ideally reach conclusions about the data that are internally valid, externally valid, and replicable. Threats to each of these domains may arise due to the intrinsic characteristics of this type of research, affecting the validity of study conclusions. The purpose of this report was to characterize analytic methods of contemporary dosimetric outcomes studies in order to determine how these threats can be addressed.

Internal validity of a study refers to the correct identification of causative relationships between variables within a study as well as how thoroughly alternative explanations for the observations are ruled out [7]. Specific threats to the internal validity of dosimetric outcomes studies include omission of variables, confounding between variables, multicollinearity, and spurious associations. Omission of variables is particularly problematic due to the possibility of unaccounted for bias within the data set. Confounding is a concern regardless of data collection methods since analysis of treatment dosimetry is nearly always post hoc. Bias within the data set can lead to confounding since the radiation exposure to an OAR is affected by other clinical factors that may, in turn, be associated with morbidity. We observed that information about non-dosimetric variables in the analysis was included in about three quarters of recently published dosimetric outcomes.

The most common analytic method to account for potential confounding between variables involves using statistical tests that simultaneously assess multiple variables, typically with multiple regression. Overall, we found that multivariate statistics were used in 226 studies, with nearly all studies using a form of either logistic regression, linear regression, or Cox regression. Variable selection for multiple regression is widely discussed, with no general consensus about the best strategy [8, 9], and several variable selection strategies are likely appropriate for dosimetric outcomes studies (so long as researchers are familiar with advantages and disadvantages of the chosen approach). The variable selection strategy used was reported by 81.4% of studies that used multivariate statistics. Nearly half of studies used a forward selection approach, where candidate variables were identified on the basis of the univariate test results and one or more multivariate models were evaluated. Backward elimination, an iterative process whereby variables are removed until a stopping rule about statistical significance or model fit is met, was the next most common strategy.

A high degree of multicollinearity among variables included in multiple regression can lead to unstable p-values for the parameter estimates and misleading interpretation of the results [10, 11]. Concerns about multicollinearity are highly relevant to dosimetric outcomes studies because DVH parameter values are highly interrelated, particularly when multiple DVH parameters are included in multiple regression tests [12]. We observed that methods to address multicollinearity were reported in fewer than half of those that used multivariate statistics. Correlation or other statistical methods to assess multicollinearity were used in 72 studies. The remaining studies assumed that DVH parameters would be collinear and took this into account when using multivariate statistics; however, this approach does not address potential multicollinearity between DVH parameters and non-dosimetric variables.

Dosimetric outcomes studies appear to be particularly at-risk for multiple comparisons problems, which famously increase risk for identifying spurious associations [13]. The possibility of multiple comparison problems in dosimetric outcomes studies is highlighted by the fact that nearly half of studies in this report assessed more than 10 DVH parameters, in addition to any clinical variables that were included. Whether or not to adjust significance thresholds is controversial and we observed this in only 44 studies [14]. Converting continuous DVH parameters into categorical variables seems a pragmatic approach for establishing treatment planning goals; however, researchers should be aware that this practice may also increase the chance of spurious findings [15]. Half of studies included in this report categorized DVH parameters using cut points, with most of these studies determining threshold values by using techniques to maximize the probability that subsequent comparisons reach statistical significance. In many prior publications, methods for categorizing continuous variables and merits of the various approaches have been reviewed. [16–18]

External validity is the degree to which the results of the study hold true outside of the study sample [7]. A classic threat to the external validity of a study is differences between the study cohort and the broader population due to selection bias or sample features. The most common source of data for dosimetric analysis was retrospective cohorts, which are recognized as very susceptible to selection bias [19]. Data from prospective cohorts and clinical trials was used in a significant minority of studies, but even though these cohorts may provide the more reliable outcomes assessment, bias remains a significant concern since dosimetric analysis is not typically a planned study endpoint.

One notable detail we observed is that most dosimetric studies adopted an exploratory approach to select DVH parameters to analyze, and reference to substantiate the selection of parameters was rare. In addition to creating challenges for maintaining internal validity (multiple comparisons, spurious associations), an exploratory approach may increase the chance of finding associations that do not generalize outside of the study population. Where possible, selecting predictors that were used in prior studies is one method to promote external validity with the advantage of also confirming the external validity of prior research findings [20, 21]. The infrequent inclusion of predictors from prior studies appears to be a missed opportunity to enhance the generalizability of findings from dosimetric outcomes studies. Similarly, when variables are dichotomized, using cut points that have been used in prior studies may reduce the chance of spurious findings, enhance validity, and make the overall body of research more cohesive.

Replicating study results is a critical aspect of the scientific process. In order to accurately replicate a study, a thorough description of the study methodology is required. Common omissions that we noted regarding study methodology were lack of details about how DVH parameters were chosen, how cut points were chosen, and the variable selection strategy for multivariate analyses. One initially surprising finding was that details about study results were missing in slightly more than one-third of studies. In other words, the methods section described analyses for which the results were either not provided, or only partially provided. On further inspection, most of these missing details were unlikely to be of interest to the casual reader but could interfere with an attempt to replicate the study findings. Examples include omission of univariate analyses and non-significant variables from multivariable models.

To better understand the impact of reporting in dosimetric outcomes studies, we assessed whether each study could be replicated based on the details provided in the manuscript and Additional file 1. Twenty-eight (8.6%) studies were classified as missing details necessary to reproduce the study results, most often because a portion of the statistical details were omitted. Though we appreciate a variety of reasons why some methodological details or results may be omitted from a study report (such as word count limits or perceived lack of interest) ensuring sufficient detail to allow for study replication is important to confirm the quality of a study.

To our knowledge, no other summary of the research methods used among dosimetric outcomes studies has been performed in the past decade. The purpose of this report was not to recommend one statistical approach over another, but rather to summarize which methods researchers are currently using and to provide a critical appraisal of issues relating to internal validity, external validity, and replicability. Perhaps the two most notable of our critical observations were the infrequent use of research quality checklists and how often details of the analytic approach and results were omitted from manuscripts. Based on the observations of recently published dosimetric outcome studies discussed above, we recommend that future studies:

1. *Reference relevant EQUATOR network statements.* The EQUATOR network provides a general framework for ensuring the quality of health research but were rarely cited by studies included in this report. The STROBE and TRIPOD statements are particularly pertinent to dosimetric outcomes studies. Citing these statements and using the accompanying checklists promotes the overall quality of the study and ensures that adequate detail is reported to replicate the study.
2. *Include non-dosimetric characteristics as predictor variables when feasible*. Considering other factors within the analysis reduces the chance of omitted variable bias. Clinical characteristics may also modify the relationship between radiation exposure and toxicity. The choice to not include other factors should be justified.
3. *Include DVH parameters and threshold values from prior literature when feasible.* Including DVH parameters that were found to be significant by others will provide external validation of prior research. Significant findings using literature based variables may have a lower chance of being spurious.
4. *Report how potential multicollinearity between predictor variables is accounted for.* DVH parameters have a high probability of collinearity which could undermine significant findings and lead to interpretation challenges. The method of assessing or preventing multicollinearity problems should be specifically addressed.
5. *Report overall model significance and goodness-of-fit when multivariate statistics are used.* Reporting model statistics is not a common practice in health research, but model statistics provide insight into how well the overall model predicts the outcome of interest, which is important for interpretation of the research findings. Given the complexity of DVH data we believe this should be standard practice.
6. *Report the entire results of all statistical comparisons.* The results of all statistical comparisons described by the study methodology should be reported. This includes reporting results for all predictor variables included in multivariate statistics. Use of  supplemental materials is appropriate for full reporting of results, particularly intermediate statistics or results that do not directly contribute to the authors’ conclusions.

We acknowledge that this focused review has its own limitations that are important to address, and published guidelines are available to help critically appraise critical reviews [22]. We addressed a focused question using a prespecified protocol in order to reduce bias and promote consistent abstraction data, minimize subjectivity, and guide interpretation. The search terms were chosen to be broad to minimize the number of DVH outcomes studies that were not captured. However, since review of all MEDLINE results returned by our search terms (138,022 results) was not feasible, we limited this review to studies published in five English language radiation oncology specialty journals which introduces a chance that our results do not generalize more broadly. Finally, we believe that our assessment of study replicability was important to report, but such an assessment is subjective depending on the experiences of the reviewer. We therefore used the conservative approach of only classifying studies as missing critical details if all reviewers independently agreed.

Dosimetric outcomes studies are valuable in radiation oncology since they represent a critical step toward improving treatment methods. Due to the complexity of relating DVH data to patient outcomes, these types of studies appear vulnerable to particular threats against internal and external validity. The recommendations provided by this report will help to address common threats and promote consistent reporting of analytic methods.

## Supplementary Information

**Additional file 1.** Review Protocol.

**Additional file 2.** Citations of works included in analysis.

## Data Availability

The datasets used and/or analyzed during the current study are available from the corresponding author on reasonable request.

## Abbreviations

OAR: Organ at risk
DVH: Dose volume histogram
QUANTEC: Quantitative analysis of normal tissue effects and complications
EQUATOR: Enhancing the quality and transparency of health research
TRIPOD: Transparent reporting of a multivariable prediction model for individual prognosis or diagnosis
STROBE: Strengthening the reporting of observational studies in epidemiology
PRISMA: Preferred reporting items for systematic reviews and meta-analyses
PMID: PubMed identifier
NTCP: Normal tissue complication probability
