# Supplementary material for: A focused review of statistical practices for relating radiation dose-volume exposure and toxicity
Source: Radiat Oncol. 2023 Mar 24;18:57. doi: 10.1186/s13014-023-02220-9 (PMC10039562; doi:10.1186/s13014-023-02220-9)
Supplement: Supplementary file 1 — Additional file 1. Review Protocol. [file 13014_2023_2220_MOESM1_ESM.docx]

**Systematic Review Protocol: Dose-Volume Histogram Analytic Methods in Radiation Oncology Research**

XXXXXX

**INTRODUCTION**

The dose-volume histogram (DVH) is method of summarizing treatment plan dosimetry that is used universally within the field of radiation oncology. The DVH summarizes the three-dimensional dose distribution within a region-of-interest (ROI). The horizontal axis of the DVH is always dose. To create a *differential DVH*, the vertical axis is the volume of the ROI receiving a given dose. To create a *cumulative DVH*, the vertical axis is the volume of the ROI receiving a given dose or more. The most commonly utilized DVH in radiation oncology research and clinical practice is the cumulative DVH. One particularly attractive aspect of DVHs is that parameters of the DVH can be statistically assessed for relationships with clinical outcomes. Relating DVH parameters to toxicity, in particular, provides critical information for radiation oncologists to guide the treatment planning process and to assess plan safety and quality. Analysis of DVH data is therefore often incorporated into clinical research studies with the goal of identifying new relationships between plan dosimetry and clinical outcomes.

While DVH calculation is a useful technique for characterizing elements of a radiation dose distribution, correlating DVH data with clinical outcomes remains statistically challenging. The 2-dimensional nature of a DVH is not suitable for traditional statistical techniques. The simplest and most commonly used methods to correlate radiation dose distribution with clinical outcomes across a population, therefore, focus on individual components of the DVH curve (e.g. maximum dose, mean dose, or volume receiving a particular dose). Thus, the 2-dimensional DVH curve is simplified into only a few elements, resulting in even further loss of dose distribution information. Different approaches to analyzing DVH data have the potential to lead to different interpretations or, in the worst case scenario, inappropriate conclusions. Despite the importance and complexity of DVH analysis, there are no universally agreed upon statistical methods or reporting standards for this research.

**REVIEW QUESTIONS**

This review seeks to characterize the various analytic methods that are used to relate DVH information to clinical outcomes and to assess the relative frequency of each approach. The specific review questions addressed are:

1. What are the most common statistical approaches used to assess the relationship between DVHs and normal tissue complication outcomes?
2. What is the relative frequency of each statistical approach?
3. For studies using multivariable statistical tests containing multiple DVH parameters, what is the frequency that co-linearity between variables is assessed?
4. For studies performing multiple analyses, what is the frequency that adjustments for multiple comparisons are made?
5. What is the frequency that goodness of fit and other model statistics are reported?
6. If threshold cut-points were used for analysis, were they arrived at statistically using the study data set or were previously published threshold values used?

**INCLUSION CRITERIA**

This review will consider all human studies that report statistical tests assessing the effect of DVH parameters on normal tissue complication outcomes.

**SEARCH STRATEGY**

1. A limited search of CINAHL and Medline to identify relevant keywords contained in the title, abstract and subject descriptors.
2. Terms identiﬁed in this way, and the synonyms used by respective databases, will be used in an extensive search of the journals selected below.

The initial search terms will be ‘Dose volume histogram’, ‘DVH’, ‘normal tissue complication’, ‘organ-at-risk’, ‘dosimetry analysis’, ‘dosimetric analysis’. We will search articles published in the 3-year interval from 2015-2019. This systematic review will be limited to journals with a primary radiation oncology audience: *International Journal of Radiation Biology * Oncology * Physics, Radiotherapy and Oncology, Practical Radiation Oncology, Radiation Oncology,* and *Acta Oncologica*.

Articles identified by the search will be assessed for inclusion based on title, abstract, key words, and other descriptors. We will obtain full copies of all articles which meet inclusion criteria for data collection and analysis.

**CRITICAL APPRAISAL**

Articles identified as meeting the inclusion criteria will be grouped into the following categories based on the source of the data used for DVH analysis: clinical trial, prospective cohort study, retrospective cohort study, and case-control study.

**DATA COLLECTION**

The methodological approach to DVH analysis in each paper will be reviewed and recorded. Specific data elements for each published manuscript are described in detail below:

- Type of radiation: External beam, brachytherapy, or both
- Description of outcome measure: The specific outcome measure as described by the study authors (e.g. CTCAE grade 2+ rectal toxicity)
- Statistical classification of outcome measure: Binary (yes/no) or continuous.
- Static or time-dependent outcome measure? Was time-to-event taken into account with respect to the outcome measure? Described as actuarial or point data.
- Source of outcomes data: Clinical trial, prospective cohort study, retrospective cohort study, matched pair study, matched cohort study, or other.
- Were NTCP calculations performed in addition to traditional DVH analysis? Yes or no.
- Statistical approach to associating DVHs with outcomes data: Describe the statistical approach, as stated in the Methods and Materials section, that was utilized to relate DVH parameters to outcome measures.
- DVH parameters analyzed: Summarize which DVH parameters were included as independent variables in the statistical analysis.
- Description of how DVH parameters were chosen for analysis: Summarize authors statements about how specific DVH elements were chosen for inclusion in the statistical analysis. If no description was provided state “Not Given”.
- Was a multivariable analysis performed? Yes or no
- What method was used to select variables for multivariable analysis? Describe how the authors chose to include variables within any multivariable models (e.g. forward selection, backward selection, clinically justified, etc.).
- Was collinearity among DVH parameters assessed for? Yes or no
- What method was used to assess for collinearity? Describe any methods used to assess for collinearity between DVH parameters. Also include if any assumptions of collinearity were made when selecting which DVH parameters within analyses.
- Were the full model results for each statistical model provided within the manuscript or as supplemental material? Yes or no. This should describe whether or not the statistical parameters of ALL variables included within EACH model are provided within the manuscript or as supplemental material. Note that statements of “models were adjusted for XXXXXX” would generally imply the entire model is NOT PRESENT unless specific details about how the model adjustments were made.
- Were multiple statistical comparisons performed for the same clinical outcome measure? Yes or no.
- Was the threshold for statistical significance adjusted for multiple comparisons? Yes or no.
- Were non-dosimetric parameters included as a covariable when assessing for association of DVH parameters with outcomes data? Yes or no.
- Were goodness-of-fit statistics reported for statistical models? Yes or no. If any goodness-of-fit evaluation was described in the Methods and Materials section please denote that here even if results were not provided.
- Were any cut-points used for specific DVH parameters? Yes or no.
- What method was used to choose cut-points? Describe the method used by the authors to determine how to convert DVH parameters from continuous variables to categorical or ordinal variables. If no description was provided state “Not Given”.
- Assuming raw data were provided, did the description of statistical methods and presentation of results provide enough information to reproduce the study results? Assuming an independent research were provided with the underlying data set and collaborated with an experienced biostatistician. Does the description of the statistical methodology provide enough information to replicate the Results that are presented? Does the Results section provide enough detail to confirm replication of the results?
- Specific concerns about reproducibility? Describe the SPECIFIC missing details that would be needed to replicate the study results.
