# Supplementary material for: A focused review of statistical practices for relating radiation dose-volume exposure and toxicity
Source: Radiat Oncol. 2023 Mar 24;18:57. doi: 10.1186/s13014-023-02220-9 (PMC10039562; doi:10.1186/s13014-023-02220-9)
Supplement: Supplementary file 2 — Additional file 2. Citations of works included in analysis. [file 13014_2023_2220_MOESM2_ESM.docx]

**References:^1-250^**

1. Beyond mean pharyngeal constrictor dose for beam path toxicity in non-target swallowing muscles: Dose-volume correlates of chronic radiation-associated dysphagia (RAD) after oropharyngeal intensity modulated radiotherapy. Radiother Oncol 2016;118(2):304-14. (In eng). DOI: 10.1016/j.radonc.2016.01.019.

2. Dose-volume correlates of mandibular osteoradionecrosis in Oropharynx cancer patients receiving intensity-modulated radiotherapy: Results from a case-matched comparison. Radiother Oncol 2017;124(2):232-239. (In eng). DOI: 10.1016/j.radonc.2017.06.026.

3. Fatigue following radiation therapy in nasopharyngeal cancer survivors: A dosimetric analysis incorporating patient report and observer rating. Radiother Oncol 2019;133:35-42. (In eng). DOI: 10.1016/j.radonc.2018.12.023.

4. Dose-volume correlates of the prevalence of patient-reported trismus in long-term survivorship after oropharyngeal IMRT: A cross-sectional dosimetric analysis. Radiother Oncol 2020;149:142-149. (In eng). DOI: 10.1016/j.radonc.2020.04.053.

5. Aarup-Kristensen S, Hansen CR, Forner L, Brink C, Eriksen JG, Johansen J. Osteoradionecrosis of the mandible after radiotherapy for head and neck cancer: risk factors and dose-volume correlations. Acta Oncol 2019;58(10):1373-1377. (In eng). DOI: 10.1080/0284186x.2019.1643037.

6. Abe T, Shirai K, Saitoh J, et al. Incidence, risk factors, and dose-volume relationship of radiation-induced rib fracture after carbon ion radiotherapy for lung cancer. Acta Oncol 2016;55(2):163-6. (In eng). DOI: 10.3109/0284186x.2015.1088169.

7. Abou Yehia Z, Mikhaeel GN, Smith G, et al. Does Bleomycin Lung Toxicity Increase the Risk of Radiation Pneumonitis in Hodgkin Lymphoma? Int J Radiat Oncol Biol Phys 2016;96(5):951-958. (In eng). DOI: 10.1016/j.ijrobp.2016.08.018.

8. Abraham AG, Usmani N, Warkentin B, et al. Dosimetric Parameters Predicting Late Small Bowel Toxicity in Patients With Rectal Cancer Receiving Neoadjuvant Chemoradiation. Pract Radiat Oncol 2021;11(1):e70-e79. (In eng). DOI: 10.1016/j.prro.2020.07.004.

9. Acharya S, Robinson CG, Michalski JM, et al. Association of 1p/19q Codeletion and Radiation Necrosis in Adult Cranial Gliomas After Proton or Photon Therapy. Int J Radiat Oncol Biol Phys 2018;101(2):334-343. (In eng). DOI: 10.1016/j.ijrobp.2018.01.099.

10. Alayed Y, Davidson M, Quon H, et al. Dosimetric predictors of toxicity and quality of life following prostate stereotactic ablative radiotherapy. Radiother Oncol 2020;144:135-140. (In eng). DOI: 10.1016/j.radonc.2019.11.017.

11. Albuquerque K, Tumati V, Lea J, et al. A Phase II Trial of Stereotactic Ablative Radiation Therapy as a Boost for Locally Advanced Cervical Cancer. Int J Radiat Oncol Biol Phys 2020;106(3):464-471. (In eng). DOI: 10.1016/j.ijrobp.2019.10.042.

12. Anderson JL, Newman NB, Anderson C, Sherry AD, Yock AD, Osmundson EC. Mean cardiopulmonary dose and vertebral marrow dose differentially predict lineage-specific leukopenia kinetics during radiotherapy for esophageal cancer. Radiother Oncol 2020;152:169-176. (In eng). DOI: 10.1016/j.radonc.2019.12.008.

13. Aoki M, Sato M, Hirose K, et al. Radiation-induced rib fracture after stereotactic body radiotherapy with a total dose of 54-56 Gy given in 9-7 fractions for patients with peripheral lung tumor: impact of maximum dose and fraction size. Radiat Oncol 2015;10:99. (In eng). DOI: 10.1186/s13014-015-0406-8.

14. Appelt AL, Bentzen SM, Jakobsen A, Vogelius IR. Dose-response of acute urinary toxicity of long-course preoperative chemoradiotherapy for rectal cancer. Acta Oncol 2015;54(2):179-86. (In eng). DOI: 10.3109/0284186x.2014.923933.

15. Atkins KM, Bitterman DS, Chaunzwa TL, et al. Statin Use, Heart Radiation Dose, and Survival in Locally Advanced Lung Cancer. Pract Radiat Oncol 2021;11(5):e459-e467. (In eng). DOI: 10.1016/j.prro.2020.12.006.

16. Barney CL, Scoville N, Allan E, et al. Radiation Dose to the Thoracic Vertebral Bodies Is Associated With Acute Hematologic Toxicities in Patients Receiving Concurrent Chemoradiation for Lung Cancer: Results of a Single-Center Retrospective Analysis. Int J Radiat Oncol Biol Phys 2018;100(3):748-755. (In eng). DOI: 10.1016/j.ijrobp.2017.11.025.

17. Barry A, McPartlin A, Lindsay P, et al. Dosimetric analysis of liver toxicity after liver metastasis stereotactic body radiation therapy. Pract Radiat Oncol 2017;7(5):e331-e337. (In eng). DOI: 10.1016/j.prro.2017.03.004.

18. Bates JE, Indelicato DJ, Morris CG, Rotondo RL, Bradley JA. Visual decline in pediatric survivors of brain tumors following radiotherapy. Acta Oncol 2020;59(10):1257-1262. (In eng). DOI: 10.1080/0284186x.2020.1803500.

19. Bauman G, Chen J, Rodrigues G, Davidson M, Warner A, Loblaw A. Extreme hypofractionation for high-risk prostate cancer: Dosimetric correlations with rectal bleeding. Pract Radiat Oncol 2017;7(6):e457-e462. (In eng). DOI: 10.1016/j.prro.2017.06.002.

20. Beasley W, Thor M, McWilliam A, et al. Image-based Data Mining to Probe Dosimetric Correlates of Radiation-induced Trismus. Int J Radiat Oncol Biol Phys 2018;102(4):1330-1338. (In eng). DOI: 10.1016/j.ijrobp.2018.05.054.

21. Bennion NR, Nowak RK, Lyden ER, Thompson RB, Li S, Lin C. Fractionated stereotactic radiation therapy for vestibular schwannomas: Dosimetric factors predictive of hearing outcomes. Pract Radiat Oncol 2016;6(5):e155-e162. (In eng). DOI: 10.1016/j.prro.2015.11.015.

22. Boonyawan K, Gomez DR, Komaki R, et al. Clinical and Dosimetric Factors Predicting Grade ≥2 Radiation Pneumonitis After Postoperative Radiotherapy for Patients With Non-Small Cell Lung Carcinoma. Int J Radiat Oncol Biol Phys 2018;101(4):919-926. (In eng). DOI: 10.1016/j.ijrobp.2018.04.012.

23. Borm KJ, Loos M, Oechsner M, et al. Acute radiodermatitis in modern adjuvant 3D conformal radiotherapy for breast cancer - the impact of dose distribution and patient related factors. Radiat Oncol 2018;13(1):218. (In eng). DOI: 10.1186/s13014-018-1160-5.

24. Breen WG, Jeans EB, Gergelis KR, et al. Ablative radiotherapy for ultracentral lung cancers: Dosimetric, geometric, and volumetric predictors of outcomes and toxicity. Radiother Oncol 2021;158:246-252. (In eng). DOI: 10.1016/j.radonc.2021.03.001.

25. Bresolin A, Faiella A, Garibaldi E, et al. Acute patient-reported intestinal toxicity in whole pelvis IMRT for prostate cancer: Bowel dose-volume effect quantification in a multicentric cohort study. Radiother Oncol 2021;158:74-82. (In eng). DOI: 10.1016/j.radonc.2021.02.026.

26. Briere TM, Krafft S, Liao Z, Martel MK. Lung Size and the Risk of Radiation Pneumonitis. Int J Radiat Oncol Biol Phys 2016;94(2):377-84. (In eng). DOI: 10.1016/j.ijrobp.2015.10.002.

27. Büchser D, Casquero F, Espinosa JM, et al. Late toxicity after single dose HDR prostate brachytherapy and EBRT for localized prostate cancer: Clinical and dosimetric predictors in a prospective cohort study. Radiother Oncol 2019;135:13-18. (In eng). DOI: 10.1016/j.radonc.2019.02.018.

28. Buranaporn P, Dankulchai P, Jaikuna T, Prasartseree T. Relation between DIR recalculated dose based CBCT and GI and GU toxicity in postoperative prostate cancer patients treated with VMAT. Radiother Oncol 2021;157:8-14. (In eng). DOI: 10.1016/j.radonc.2020.12.036.

29. Bütof R, Löck S, Soliman M, et al. Dose-volume predictors of early esophageal toxicity in non-small cell lung cancer patients treated with accelerated-hyperfractionated radiotherapy. Radiother Oncol 2020;143:44-50. (In eng). DOI: 10.1016/j.radonc.2019.11.002.

30. Cao J, Zhang X, Jiang B, et al. Intensity-modulated proton therapy for oropharyngeal cancer reduces rates of late xerostomia. Radiother Oncol 2021;160:32-39. (In eng). DOI: 10.1016/j.radonc.2021.03.036.

31. Casares-Magaz O, Bülow S, Pettersson NJ, et al. High accumulated doses to the inferior rectum are associated with late gastro-intestinal toxicity in a case-control study of prostate cancer patients treated with radiotherapy. Acta Oncol 2019;58(10):1543-1546. (In eng). DOI: 10.1080/0284186x.2019.1632476.

32. Cella L, D'Avino V, Palma G, et al. Modeling the risk of radiation-induced lung fibrosis: Irradiated heart tissue is as important as irradiated lung. Radiother Oncol 2015;117(1):36-43. (In eng). DOI: 10.1016/j.radonc.2015.07.051.

33. Cella L, Oh JH, Deasy JO, et al. Predicting radiation-induced valvular heart damage. Acta Oncol 2015;54(10):1796-804. (In eng). DOI: 10.3109/0284186x.2015.1016624.

34. Chadha AS, Liu G, Chen HC, et al. Does Unintentional Splenic Radiation Predict Outcomes After Pancreatic Cancer Radiation Therapy? Int J Radiat Oncol Biol Phys 2017;97(2):323-332. (In eng). DOI: 10.1016/j.ijrobp.2016.10.046.

35. Chasseray M, Dissaux G, Bourbonne V, et al. Dose to the penile bulb and individual patient anatomy are predictive of erectile dysfunction in men treated with (125)I low dose rate brachytherapy for localized prostate cancer. Acta Oncol 2019;58(7):1029-1035. (In eng). DOI: 10.1080/0284186x.2019.1574981.

36. Chaudhuri AA, Binkley MS, Rigdon J, et al. Pre-treatment non-target lung FDG-PET uptake predicts symptomatic radiation pneumonitis following Stereotactic Ablative Radiotherapy (SABR). Radiother Oncol 2016;119(3):454-60. (In eng). DOI: 10.1016/j.radonc.2016.05.007.

37. Chen AM, Yoshizaki T, Velez MA, Mikaeilian AG, Hsu S, Cao M. Tolerance of the Brachial Plexus to High-Dose Reirradiation. Int J Radiat Oncol Biol Phys 2017;98(1):83-90. (In eng). DOI: 10.1016/j.ijrobp.2017.01.244.

38. Chen D, Patel RR, Verma V, et al. Interaction between lymphopenia, radiotherapy technique, dosimetry, and survival outcomes in lung cancer patients receiving combined immunotherapy and radiotherapy. Radiother Oncol 2020;150:114-120. (In eng). DOI: 10.1016/j.radonc.2020.05.051.

39. Chera BS, Fried D, Price A, et al. Dosimetric Predictors of Patient-Reported Xerostomia and Dysphagia With Deintensified Chemoradiation Therapy for HPV-Associated Oropharyngeal Squamous Cell Carcinoma. Int J Radiat Oncol Biol Phys 2017;98(5):1022-1027. (In eng). DOI: 10.1016/j.ijrobp.2017.03.034.

40. Chipko C, Ojwang J, Gharai LR, Deng X, Mukhopadhyay N, Weiss E. Characterization of Chest Wall Toxicity During Long-term Follow Up After Thoracic Stereotactic Body Radiation Therapy. Pract Radiat Oncol 2019;9(3):e338-e346. (In eng). DOI: 10.1016/j.prro.2019.01.012.

41. Cho WK, Oh D, Kim HK, et al. Dosimetric predictors for postoperative pulmonary complications in esophageal cancer following neoadjuvant chemoradiotherapy and surgery. Radiother Oncol 2019;133:87-92. (In eng). DOI: 10.1016/j.radonc.2019.01.005.

42. Choi SH, Chang JS, Byun HK, et al. Risk of Hypothyroidism in Women After Radiation Therapy for Breast Cancer. Int J Radiat Oncol Biol Phys 2021;110(2):462-472. (In eng). DOI: 10.1016/j.ijrobp.2020.12.047.

43. Chow JCH, Cheung KM, Au KH, et al. Radiation-induced hypoglossal nerve palsy after definitive radiotherapy for nasopharyngeal carcinoma: Clinical predictors and dose-toxicity relationship. Radiother Oncol 2019;138:93-98. (In eng). DOI: 10.1016/j.radonc.2019.06.011.

44. Christophides D, Appelt AL, Gusnanto A, Lilley J, Sebag-Montefiore D. Method for Automatic Selection of Parameters in Normal Tissue Complication Probability Modeling. Int J Radiat Oncol Biol Phys 2018;101(3):704-712. (In eng). DOI: 10.1016/j.ijrobp.2018.02.152.

45. Chung SY, Oh J, Chang JS, et al. Risk of Cardiac Disease in Patients With Breast Cancer: Impact of Patient-Specific Factors and Individual Heart Dose From Three-Dimensional Radiation Therapy Planning. Int J Radiat Oncol Biol Phys 2021;110(2):473-481. (In eng). DOI: 10.1016/j.ijrobp.2020.12.053.

46. Cohen-Cutler S, Wong K, Mena V, et al. Hearing Loss Risk in Pediatric Patients Treated with Cranial Irradiation and Cisplatin-Based Chemotherapy. Int J Radiat Oncol Biol Phys 2021;110(5):1488-1495. (In eng). DOI: 10.1016/j.ijrobp.2021.02.050.

47. Colaco RJ, Hoppe BS, Flampouri S, et al. Rectal toxicity after proton therapy for prostate cancer: an analysis of outcomes of prospective studies conducted at the university of Florida Proton Therapy Institute. Int J Radiat Oncol Biol Phys 2015;91(1):172-81. (In eng). DOI: 10.1016/j.ijrobp.2014.08.353.

48. Connor M, Karunamuni R, McDonald C, et al. Regional susceptibility to dose-dependent white matter damage after brain radiotherapy. Radiother Oncol 2017;123(2):209-217. (In eng). DOI: 10.1016/j.radonc.2017.04.006.

49. Crook JM, Zhang P, Pisansky TM, et al. A Prospective Phase 2 Trial of Transperineal Ultrasound-Guided Brachytherapy for Locally Recurrent Prostate Cancer After External Beam Radiation Therapy (NRG Oncology/RTOG-0526). Int J Radiat Oncol Biol Phys 2019;103(2):335-343. (In eng). DOI: 10.1016/j.ijrobp.2018.09.039.

50. de Groot C, Beukema JC, Langendijk JA, et al. Radiation-Induced Myocardial Fibrosis in Long-Term Esophageal Cancer Survivors. Int J Radiat Oncol Biol Phys 2021;110(4):1013-1021. (In eng). DOI: 10.1016/j.ijrobp.2021.02.007.

51. De Leo AN, Holtzman AL, Ho MW, et al. Vision loss following high-dose proton-based radiotherapy for skull-base chordoma and chondrosarcoma. Radiother Oncol 2021;158:125-130. (In eng). DOI: 10.1016/j.radonc.2021.02.012.

52. Dean JA, Wong KH, Gay H, et al. Functional Data Analysis Applied to Modeling of Severe Acute Mucositis and Dysphagia Resulting From Head and Neck Radiation Therapy. Int J Radiat Oncol Biol Phys 2016;96(4):820-831. (In eng). DOI: 10.1016/j.ijrobp.2016.08.013.

53. Dean JA, Wong KH, Welsh LC, et al. Normal tissue complication probability (NTCP) modelling using spatial dose metrics and machine learning methods for severe acute oral mucositis resulting from head and neck radiotherapy. Radiother Oncol 2016;120(1):21-7. (In eng). DOI: 10.1016/j.radonc.2016.05.015.

54. Deek MP, Benenati B, Kim S, et al. Thoracic Vertebral Body Irradiation Contributes to Acute Hematologic Toxicity During Chemoradiation Therapy for Non-Small Cell Lung Cancer. Int J Radiat Oncol Biol Phys 2016;94(1):147-154. (In eng). DOI: 10.1016/j.ijrobp.2015.09.022.

55. Deek MP, Nagarajan S, Kim S, et al. Clinical characteristics and dose-volume histogram parameters associated with the development of pleural effusions in non-small cell lung cancer patients treated with chemoradiation therapy. Acta Oncol 2016;55(8):1029-35. (In eng). DOI: 10.1080/0284186x.2016.1176248.

56. Defraene G, Schuit E, De Ruysscher D. Development and internal validation of a multinomial NTCP model for the severity of acute dyspnea after radiotherapy for lung cancer. Radiother Oncol 2019;136:176-184. (In eng). DOI: 10.1016/j.radonc.2019.03.034.

57. Demissei BG, Freedman G, Feigenberg SJ, et al. Early Changes in Cardiovascular Biomarkers with Contemporary Thoracic Radiation Therapy for Breast Cancer, Lung Cancer, and Lymphoma. Int J Radiat Oncol Biol Phys 2019;103(4):851-860. (In eng). DOI: 10.1016/j.ijrobp.2018.11.013.

58. Deville C, Jr., Jain A, Hwang WT, et al. Initial report of the genitourinary and gastrointestinal toxicity of post-prostatectomy proton therapy for prostate cancer patients undergoing adjuvant or salvage radiotherapy. Acta Oncol 2018;57(11):1506-1514. (In eng). DOI: 10.1080/0284186x.2018.1487583.

59. Din SU, Williams EL, Jackson A, et al. Impact of Fractionation and Dose in a Multivariate Model for Radiation-Induced Chest Wall Pain. Int J Radiat Oncol Biol Phys 2015;93(2):418-24. (In eng). DOI: 10.1016/j.ijrobp.2015.06.014.

60. Dinh TT, Lee HJ, Jr., Macomber MW, et al. Rectal Hydrogel Spacer Improves Late Gastrointestinal Toxicity Compared to Rectal Balloon Immobilization After Proton Beam Radiation Therapy for Localized Prostate Cancer: A Retrospective Observational Study. Int J Radiat Oncol Biol Phys 2020;108(3):635-643. (In eng). DOI: 10.1016/j.ijrobp.2020.01.026.

61. Dréan G, Acosta O, Ospina JD, et al. Identification of a rectal subregion highly predictive of rectal bleeding in prostate cancer IMRT. Radiother Oncol 2016;119(3):388-97. (In eng). DOI: 10.1016/j.radonc.2016.04.023.

62. Duijm M, Tekatli H, Oomen-de Hoop E, et al. Esophagus toxicity after stereotactic and hypofractionated radiotherapy for central lung tumors: Normal tissue complication probability modeling. Radiother Oncol 2018;127(2):233-238. (In eng). DOI: 10.1016/j.radonc.2018.02.004.

63. Duijm M, van der Voort van Zyp NC, van de Vaart P, et al. Predicting High-Grade Esophagus Toxicity After Treating Central Lung Tumors With Stereotactic Radiation Therapy Using a Normal Tissue Complication Probability Model. Int J Radiat Oncol Biol Phys 2020;106(1):73-81. (In eng). DOI: 10.1016/j.ijrobp.2019.08.059.

64. Dutz A, Agolli L, Baumann M, et al. Early and late side effects, dosimetric parameters and quality of life after proton beam therapy and IMRT for prostate cancer: a matched-pair analysis. Acta Oncol 2019;58(6):916-925. (In eng). DOI: 10.1080/0284186x.2019.1581373.

65. Dutz A, Lühr A, Agolli L, et al. Modelling of late side-effects following cranial proton beam therapy. Radiother Oncol 2021;157:15-23. (In eng). DOI: 10.1016/j.radonc.2021.01.004.

66. Dyk P, Weiner A, Badiyan S, Myerson R, Parikh P, Olsen J. Effect of high-dose stereotactic body radiation therapy on liver function in the treatment of primary and metastatic liver malignancies using the Child-Pugh score classification system. Pract Radiat Oncol 2015;5(3):176-182. (In eng). DOI: 10.1016/j.prro.2014.09.007.

67. Ebert MA, Foo K, Haworth A, et al. Gastrointestinal dose-histogram effects in the context of dose-volume-constrained prostate radiation therapy: analysis of data from the RADAR prostate radiation therapy trial. Int J Radiat Oncol Biol Phys 2015;91(3):595-603. (In eng). DOI: 10.1016/j.ijrobp.2014.11.015.

68. El-Fayech C, Haddy N, Allodji RS, et al. Cerebrovascular Diseases in Childhood Cancer Survivors: Role of the Radiation Dose to Willis Circle Arteries. Int J Radiat Oncol Biol Phys 2017;97(2):278-286. (In eng). DOI: 10.1016/j.ijrobp.2016.10.015.

69. Esiashvili N, Lu X, Ulin K, et al. Higher Reported Lung Dose Received During Total Body Irradiation for Allogeneic Hematopoietic Stem Cell Transplantation in Children With Acute Lymphoblastic Leukemia Is Associated With Inferior Survival: A Report from the Children's Oncology Group. Int J Radiat Oncol Biol Phys 2019;104(3):513-521. (In eng). DOI: 10.1016/j.ijrobp.2019.02.034.

70. Everitt S, Duffy M, Bressel M, et al. Association of oesophageal radiation dose volume metrics, neutropenia and acute radiation oesophagitis in patients receiving chemoradiotherapy for non-small cell lung cancer. Radiat Oncol 2016;11:20. (In eng). DOI: 10.1186/s13014-016-0596-8.

71. Fang P, Swanick CW, Pezzi TA, et al. Outcomes and toxicity following high-dose radiation therapy in 15 fractions for non-small cell lung cancer. Pract Radiat Oncol 2017;7(6):433-441. (In eng). DOI: 10.1016/j.prro.2017.03.005.

72. Fargeas A, Acosta O, Ospina Arrango JD, et al. Independent component analysis for rectal bleeding prediction following prostate cancer radiotherapy. Radiother Oncol 2018;126(2):263-269. (In eng). DOI: 10.1016/j.radonc.2017.11.011.

73. Farr KP, Kallehauge JF, Møller DS, et al. Inclusion of functional information from perfusion SPECT improves predictive value of dose-volume parameters in lung toxicity outcome after radiotherapy for non-small cell lung cancer: A prospective study. Radiother Oncol 2015;117(1):9-16. (In eng). DOI: 10.1016/j.radonc.2015.08.005.

74. Farr KP, Khalil AA, Møller DS, et al. Time and dose-related changes in lung perfusion after definitive radiotherapy for NSCLC. Radiother Oncol 2018;126(2):307-311. (In eng). DOI: 10.1016/j.radonc.2017.11.008.

75. Faruqi S, Ruschin M, Soliman H, et al. Adverse Radiation Effect After Hypofractionated Stereotactic Radiosurgery in 5 Daily Fractions for Surgical Cavities and Intact Brain Metastases. Int J Radiat Oncol Biol Phys 2020;106(4):772-779. (In eng). DOI: 10.1016/j.ijrobp.2019.12.002.

76. Ferris MJ, Zhong J, Switchenko JM, et al. Brainstem dose is associated with patient-reported acute fatigue in head and neck cancer radiation therapy. Radiother Oncol 2018;126(1):100-106. (In eng). DOI: 10.1016/j.radonc.2017.08.008.

77. Fokdal L, Tanderup K, Pötter R, et al. Risk Factors for Ureteral Stricture After Radiochemotherapy Including Image Guided Adaptive Brachytherapy in Cervical Cancer: Results From the EMBRACE Studies. Int J Radiat Oncol Biol Phys 2019;103(4):887-894. (In eng). DOI: 10.1016/j.ijrobp.2018.11.006.

78. Franzese C, Fogliata A, Clerici E, et al. Toxicity profile and early clinical outcome for advanced head and neck cancer patients treated with simultaneous integrated boost and volumetric modulated arc therapy. Radiat Oncol 2015;10:224. (In eng). DOI: 10.1186/s13014-015-0535-0.

79. Frelinghuysen M, Schillemans W, Hol L, Verhoef C, Hoogeman M, Nuyttens JJ. Acute toxicity of the bowel after stereotactic robotic radiotherapy for abdominopelvic oligometastases. Acta Oncol 2018;57(4):480-484. (In eng). DOI: 10.1080/0284186x.2017.1378432.

80. Garant A, Spears G, Routman D, et al. A Multi-Institutional Analysis of Radiation Dosimetric Predictors of Toxicity After Trimodality Therapy for Esophageal Cancer. Pract Radiat Oncol 2021;11(4):e415-e425. (In eng). DOI: 10.1016/j.prro.2021.01.004.

81. Garbacz M, Cordoni FG, Durante M, et al. Study of relationship between dose, LET and the risk of brain necrosis after proton therapy for skull base tumors. Radiother Oncol 2021;163:143-149. (In eng). DOI: 10.1016/j.radonc.2021.08.015.

82. García-Consuegra A, Gimeno Morales M, Cambeiro M, et al. Dose volume histogram constraints in patients with head and neck cancer treated with surgery and adjuvant HDR brachytherapy: A proposal of the head and neck and skin GEC ESTRO Working group. Radiother Oncol 2021;154:128-134. (In eng). DOI: 10.1016/j.radonc.2020.09.015.

83. Gebhardt BJ, Vargo JA, Ling D, et al. Carotid Dosimetry and the Risk of Carotid Blowout Syndrome After Reirradiation With Head and Neck Stereotactic Body Radiation Therapy. Int J Radiat Oncol Biol Phys 2018;101(1):195-200. (In eng). DOI: 10.1016/j.ijrobp.2017.11.045.

84. Gebre-Medhin M, Haghanegi M, Robért L, Kjellén E, Nilsson P. Dose-volume analysis of radiation-induced trismus in head and neck cancer patients. Acta Oncol 2016;55(11):1313-1317. (In eng). DOI: 10.1080/0284186x.2016.1221129.

85. Gensheimer MF, Nyflot M, Laramore GE, Liao JJ, Parvathaneni U. Contribution of submandibular gland and swallowing structure sparing to post-radiation therapy PEG dependence in oropharynx cancer patients treated with split-neck IMRT technique. Radiat Oncol 2016;11(1):151. (In eng). DOI: 10.1186/s13014-016-0726-3.

86. Gentile MS, Yeap BY, Paganetti H, et al. Brainstem Injury in Pediatric Patients With Posterior Fossa Tumors Treated With Proton Beam Therapy and Associated Dosimetric Factors. Int J Radiat Oncol Biol Phys 2018;100(3):719-729. (In eng). DOI: 10.1016/j.ijrobp.2017.11.026.

87. Gomez CL, Xu X, Qi XS, et al. Dosimetric parameters predict short-term quality-of-life outcomes for patients receiving stereotactic body radiation therapy for prostate cancer. Pract Radiat Oncol 2015;5(4):257-62. (In eng). DOI: 10.1016/j.prro.2015.01.006.

88. Graffeo CS, Link MJ, Brown PD, Young WF, Jr., Pollock BE. Hypopituitarism After Single-Fraction Pituitary Adenoma Radiosurgery: Dosimetric Analysis Based on Patients Treated Using Contemporary Techniques. Int J Radiat Oncol Biol Phys 2018;101(3):618-623. (In eng). DOI: 10.1016/j.ijrobp.2018.02.169.

89. Grant JD, Shirvani SM, Tang C, et al. Incidence and predictors of severe acute esophagitis and subsequent esophageal stricture in patients treated with accelerated hyperfractionated chemoradiation for limited-stage small cell lung cancer. Pract Radiat Oncol 2015;5(4):e383-91. (In eng). DOI: 10.1016/j.prro.2015.01.005.

90. Gross JP, Lynch CM, Flores AM, et al. Determining the Organ at Risk for Lymphedema After Regional Nodal Irradiation in Breast Cancer. Int J Radiat Oncol Biol Phys 2019;105(3):649-658. (In eng). DOI: 10.1016/j.ijrobp.2019.06.2509.

91. Guo W, Hui X, Alfaifi S, et al. Preoperative contralateral lung radiation dose is associated with postoperative pulmonary toxicity in patients with locally advanced non-small cell lung cancer treated with trimodality therapy. Pract Radiat Oncol 2018;8(4):e239-e248. (In eng). DOI: 10.1016/j.prro.2018.01.004.

92. Hahn E, Jiang H, Ng A, et al. Late Cardiac Toxicity After Mediastinal Radiation Therapy for Hodgkin Lymphoma: Contributions of Coronary Artery and Whole Heart Dose-Volume Variables to Risk Prediction. Int J Radiat Oncol Biol Phys 2017;98(5):1116-1123. (In eng). DOI: 10.1016/j.ijrobp.2017.03.026.

93. Harder EM, Park HS, Chen ZJ, Decker RH. Pulmonary dose-volume predictors of radiation pneumonitis following stereotactic body radiation therapy. Pract Radiat Oncol 2016;6(6):e353-e359. (In eng). DOI: 10.1016/j.prro.2016.01.015.

94. Hawkins PG, Boonstra PS, Hobson ST, et al. Radiation-induced lung toxicity in non-small-cell lung cancer: Understanding the interactions of clinical factors and cytokines with the dose-toxicity relationship. Radiother Oncol 2017;125(1):66-72. (In eng). DOI: 10.1016/j.radonc.2017.09.005.

95. Hayakawa T, Kawakami S, Soda I, et al. Dosimetric factors associated with long-term patient-reported outcomes after definitive radiotherapy of patients with head and neck cancer. Radiat Oncol 2019;14(1):221. (In eng). DOI: 10.1186/s13014-019-1429-3.

96. Hayashi K, Yamamoto N, Karube M, et al. Prognostic analysis of radiation pneumonitis: carbon-ion radiotherapy in patients with locally advanced lung cancer. Radiat Oncol 2017;12(1):91. (In eng). DOI: 10.1186/s13014-017-0830-z.

97. Heilemann G, Fetty L, Blaickner M, et al. Retina dose as a predictor for visual acuity loss in (106)Ru eye plaque brachytherapy of uveal melanomas. Radiother Oncol 2018;127(3):379-384. (In eng). DOI: 10.1016/j.radonc.2017.11.010.

98. Hol MLF, Indelicato DJ, Rotondo RL, et al. Dose-Effect Analysis of Early Changes in Orbital Bone Morphology After Radiation Therapy for Rhabdomyosarcoma. Pract Radiat Oncol 2020;10(1):53-58. (In eng). DOI: 10.1016/j.prro.2019.10.002.

99. Holliday EB, Esmaeli B, Pinckard J, et al. A Multidisciplinary Orbit-Sparing Treatment Approach That Includes Proton Therapy for Epithelial Tumors of the Orbit and Ocular Adnexa. Int J Radiat Oncol Biol Phys 2016;95(1):344-352. (In eng). DOI: 10.1016/j.ijrobp.2015.08.008.

100. Huang H, Roberson J, Hou W, et al. NTCP model for hypothyroidism after supraclavicular-directed radiation therapy for breast cancer. Radiother Oncol 2021;154:87-92. (In eng). DOI: 10.1016/j.radonc.2020.09.003.

101. Huang J, DeWees TA, Badiyan SN, et al. Clinical and Dosimetric Predictors of Acute Severe Lymphopenia During Radiation Therapy and Concurrent Temozolomide for High-Grade Glioma. Int J Radiat Oncol Biol Phys 2015;92(5):1000-1007. (In eng). DOI: 10.1016/j.ijrobp.2015.04.005.

102. Huang J, Gu F, Ji T, Zhao J, Li G. Pelvic bone marrow sparing intensity modulated radiotherapy reduces the incidence of the hematologic toxicity of patients with cervical cancer receiving concurrent chemoradiotherapy: a single-center prospective randomized controlled trial. Radiat Oncol 2020;15(1):180. (In eng). DOI: 10.1186/s13014-020-01606-3.

103. Huang J, Kong FF, Oei RW, Zhai RP, Hu CS, Ying HM. Dosimetric predictors of temporal lobe injury after intensity-modulated radiotherapy for T4 nasopharyngeal carcinoma: a competing risk study. Radiat Oncol 2019;14(1):31. (In eng). DOI: 10.1186/s13014-019-1229-9.

104. Huang XD, Li YC, Chen FP, et al. Evolution and Dosimetric Analysis of Magnetic Resonance Imaging-Detected Brain Stem Injury After Intensity Modulated Radiation Therapy in Nasopharyngeal Carcinoma. Int J Radiat Oncol Biol Phys 2019;105(1):124-131. (In eng). DOI: 10.1016/j.ijrobp.2019.04.032.

105. Huang Y, Chen SW, Fan CC, Ting LL, Kuo CC, Chiou JF. Clinical parameters for predicting radiation-induced liver disease after intrahepatic reirradiation for hepatocellular carcinoma. Radiat Oncol 2016;11(1):89. (In eng). DOI: 10.1186/s13014-016-0663-1.

106. Inaba K, Okamoto H, Wakita A, et al. Long-term observations of radiation-induced creatinine clearance reduction and renal parenchymal volume atrophy. Radiother Oncol 2016;120(1):145-9. (In eng). DOI: 10.1016/j.radonc.2016.04.022.

107. Jacobse JN, Duane FK, Boekel NB, et al. Radiation Dose-Response for Risk of Myocardial Infarction in Breast Cancer Survivors. Int J Radiat Oncol Biol Phys 2019;103(3):595-604. (In eng). DOI: 10.1016/j.ijrobp.2018.10.025.

108. Jalali R, Maitre M, Gupta T, et al. Dose-Constraint Model to Predict Neuroendocrine Dysfunction in Young Patients With Brain Tumors: Data From a Prospective Study. Pract Radiat Oncol 2019;9(4):e362-e371. (In eng). DOI: 10.1016/j.prro.2019.02.011.

109. Jang BS, Cha MJ, Kim HJ, et al. Heart substructural dosimetric parameters and risk of cardiac events after definitive chemoradiotherapy for stage III non-small cell lung cancer. Radiother Oncol 2020;152:126-132. (In eng). DOI: 10.1016/j.radonc.2020.09.050.

110. Jang JY, Kim SS, Song SY, Kim YJ, Kim SW, Choi EK. Radiation pneumonitis in patients with non-small-cell lung cancer receiving chemoradiotherapy and an immune checkpoint inhibitor: a retrospective study. Radiat Oncol 2021;16(1):231. (In eng). DOI: 10.1186/s13014-021-01930-2.

111. Jiang Y, Ji Z, Guo F, et al. Side effects of CT-guided implantation of (125)I seeds for recurrent malignant tumors of the head and neck assisted by 3D printing non co-planar template. Radiat Oncol 2018;13(1):18. (In eng). DOI: 10.1186/s13014-018-0959-4.

112. Joseph N, McWilliam A, Kennedy J, et al. Post-treatment lymphocytopaenia, integral body dose and overall survival in lung cancer patients treated with radical radiotherapy. Radiother Oncol 2019;135:115-119. (In eng). DOI: 10.1016/j.radonc.2019.03.008.

113. Journy N, Schonfeld SJ, Hauptmann M, et al. Dose-volume effects of breast cancer radiation therapy on the risk of second oesophageal cancer. Radiother Oncol 2020;151:33-39. (In eng). DOI: 10.1016/j.radonc.2020.07.022.

114. Julie DA, Oh JH, Apte AP, et al. Predictors of acute toxicities during definitive chemoradiation using intensity-modulated radiotherapy for anal squamous cell carcinoma. Acta Oncol 2016;55(2):208-16. (In eng). DOI: 10.3109/0284186x.2015.1043396.

115. Kaae JK, Johnsen L, Hansen CR, Kristensen MH, Brink C, Eriksen JG. Relationship between patient and physician-rated xerostomia and dose distribution to the oral cavity and salivary glands for head and neck cancer patients after radiotherapy. Acta Oncol 2019;58(10):1366-1372. (In eng). DOI: 10.1080/0284186x.2019.1627413.

116. Kamal M, Mohamed ASR, Volpe S, et al. Radiotherapy dose-volume parameters predict videofluoroscopy-detected dysphagia per DIGEST after IMRT for oropharyngeal cancer: Results of a prospective registry. Radiother Oncol 2018;128(3):442-451. (In eng). DOI: 10.1016/j.radonc.2018.06.013.

117. Katsoulakis E, Jackson A, Cox B, Lovelock M, Yamada Y. A Detailed Dosimetric Analysis of Spinal Cord Tolerance in High-Dose Spine Radiosurgery. Int J Radiat Oncol Biol Phys 2017;99(3):598-607. (In eng). DOI: 10.1016/j.ijrobp.2017.05.053.

118. Kawamura M, Yoshimura M, Asada H, Nakamura M, Matsuo Y, Mizowaki T. A scoring system predicting acute radiation dermatitis in patients with head and neck cancer treated with intensity-modulated radiotherapy. Radiat Oncol 2019;14(1):14. (In eng). DOI: 10.1186/s13014-019-1215-2.

119. Kazemzadeh N, Modiri A, Samanta S, et al. Virtual Bronchoscopy-Guided Treatment Planning to Map and Mitigate Radiation-Induced Airway Injury in Lung SAbR. Int J Radiat Oncol Biol Phys 2018;102(1):210-218. (In eng). DOI: 10.1016/j.ijrobp.2018.04.060.

120. Kim D, Nam J, Kim W, et al. Radiotherapy dose-volume parameters predict facial lymphedema after concurrent chemoradiation for nasopharyngeal carcinoma. Radiat Oncol 2021;16(1):172. (In eng). DOI: 10.1186/s13014-021-01901-7.

121. Kindts I, Defraene G, Laenen A, et al. Development of a normal tissue complication probability model for late unfavourable aesthetic outcome after breast-conserving therapy. Acta Oncol 2018;57(7):916-923. (In eng). DOI: 10.1080/0284186x.2018.1461926.

122. Kindts I, Defraene G, Petillion S, et al. Validation of a normal tissue complication probability model for late unfavourable aesthetic outcome after breast-conserving therapy. Acta Oncol 2019;58(4):448-455. (In eng). DOI: 10.1080/0284186x.2018.1548775.

123. Koëter M, Kathiravetpillai N, Gooszen JA, et al. Influence of the Extent and Dose of Radiation on Complications After Neoadjuvant Chemoradiation and Subsequent Esophagectomy With Gastric Tube Reconstruction With a Cervical Anastomosis. Int J Radiat Oncol Biol Phys 2017;97(4):813-821. (In eng). DOI: 10.1016/j.ijrobp.2016.11.054.

124. Koëter M, van der Sangen MJ, Hurkmans CW, Luyer MD, Rutten HJ, Nieuwenhuijzen GA. Radiation dose does not influence anastomotic complications in patients with esophageal cancer treated with neoadjuvant chemoradiation and transhiatal esophagectomy. Radiat Oncol 2015;10:59. (In eng). DOI: 10.1186/s13014-015-0361-4.

125. Kole TP, Tong M, Wu B, et al. Late urinary toxicity modeling after stereotactic body radiotherapy (SBRT) in the definitive treatment of localized prostate cancer. Acta Oncol 2016;55(1):52-8. (In eng). DOI: 10.3109/0284186x.2015.1037011.

126. Kubo N, Kubota Y, Kawamura H, et al. Dosimetric parameters predictive of nasolacrimal duct obstruction after carbon-ion radiotherapy for head and neck carcinoma. Radiother Oncol 2019;141:72-77. (In eng). DOI: 10.1016/j.radonc.2019.07.022.

127. Kubota H, Miyawaki D, Mukumoto N, et al. Risk factors for osteoradionecrosis of the jaw in patients with head and neck squamous cell carcinoma. Radiat Oncol 2021;16(1):1. (In eng). DOI: 10.1186/s13014-020-01701-5.

128. Kunogi H, Yamaguchi N, Terao Y, Sasai K. Dosimetric predictors of nephrotoxicity in patients receiving extended-field radiation therapy for gynecologic cancer. Radiat Oncol 2021;16(1):25. (In eng). DOI: 10.1186/s13014-021-01755-z.

129. Kuo AH, Ancukiewicz M, Kozak KR, Yock TI, Padera TP. Cardiac and inflammatory biomarkers do not correlate with volume of heart or lung receiving radiation. Radiat Oncol 2015;10:5. (In eng). DOI: 10.1186/s13014-014-0324-1.

130. Lamba N, Bussiere MR, Niemierko A, et al. Hypopituitarism After Cranial Irradiation for Meningiomas: A Single-Institution Experience. Pract Radiat Oncol 2019;9(3):e266-e273. (In eng). DOI: 10.1016/j.prro.2019.01.009.

131. Laugaard Lorenzen E, Christian Rehammar J, Jensen MB, Ewertz M, Brink C. Radiation-induced risk of ischemic heart disease following breast cancer radiotherapy in Denmark, 1977-2005. Radiother Oncol 2020;152:103-110. (In eng). DOI: 10.1016/j.radonc.2020.08.007.

132. Le Fèvre C, Cheng X, Loit MP, et al. Role of hippocampal location and radiation dose in glioblastoma patients with hippocampal atrophy. Radiat Oncol 2021;16(1):112. (In eng). DOI: 10.1186/s13014-021-01835-0.

133. Lee AY, Golden DW, Bazan JG, et al. Hematologic Nadirs During Chemoradiation for Anal Cancer: Temporal Characterization and Dosimetric Predictors. Int J Radiat Oncol Biol Phys 2017;97(2):306-312. (In eng). DOI: 10.1016/j.ijrobp.2016.10.010.

134. Lee HJ, Jr., Stacey A, Klesert TR, et al. Corneal Substructure Dosimetry Predicts Corneal Toxicity in Patients With Uveal Melanoma Treated With Proton Beam Therapy. Int J Radiat Oncol Biol Phys 2019;104(2):374-382. (In eng). DOI: 10.1016/j.ijrobp.2019.02.005.

135. Lee MY, Ouyang Z, LaHurd D, et al. A Volumetric Dosimetry Analysis of Vertebral Body Fracture Risk After Single Fraction Spine Stereotactic Body Radiation Therapy. Pract Radiat Oncol 2021;11(6):480-487. (In eng). DOI: 10.1016/j.prro.2021.07.004.

136. Lehrer EJ, Snyder MH, Desai BD, et al. Clinical and radiographic adverse events after Gamma Knife radiosurgery for brainstem lesions: A dosimetric analysis. Radiother Oncol 2020;147:200-209. (In eng). DOI: 10.1016/j.radonc.2020.05.010.

137. Li F, Liu H, Wu H, Liang S, Xu Y. Risk factors for radiation pneumonitis in lung cancer patients with subclinical interstitial lung disease after thoracic radiation therapy. Radiat Oncol 2021;16(1):70. (In eng). DOI: 10.1186/s13014-021-01798-2.

138. Li F, Zhou Z, Wu A, et al. Preexisting radiological interstitial lung abnormalities are a risk factor for severe radiation pneumonitis in patients with small-cell lung cancer after thoracic radiation therapy. Radiat Oncol 2018;13(1):82. (In eng). DOI: 10.1186/s13014-018-1030-1.

139. Li PC, Liebsch NJ, Niemierko A, et al. Radiation tolerance of the optic pathway in patients treated with proton and photon radiotherapy. Radiother Oncol 2019;131:112-119. (In eng). DOI: 10.1016/j.radonc.2018.12.007.

140. Liang X, Bradley JA, Zheng D, et al. Prognostic factors of radiation dermatitis following passive-scattering proton therapy for breast cancer. Radiat Oncol 2018;13(1):72. (In eng). DOI: 10.1186/s13014-018-1004-3.

141. Lin G, Xiao H, Zeng Z, et al. Constraints for symptomatic radiation pneumonitis of helical tomotherapy hypofractionated simultaneous multitarget radiotherapy for pulmonary metastasis from hepatocellular carcinoma. Radiother Oncol 2017;123(2):246-250. (In eng). DOI: 10.1016/j.radonc.2017.02.015.

142. Lin JB, Hung LC, Cheng CY, et al. Prognostic significance of lung radiation dose in patients with esophageal cancer treated with neoadjuvant chemoradiotherapy. Radiat Oncol 2019;14(1):85. (In eng). DOI: 10.1186/s13014-019-1283-3.

143. Lind H, Alevronta E, Steineck G, et al. Defecation into clothing without forewarning and mean radiation dose to bowel and anal-sphincter among gynecological cancer survivors. Acta Oncol 2016;55(11):1285-1293. (In eng). DOI: 10.1080/0284186x.2016.1176247.

144. Ling DC, Flickinger JC, Burton SA, et al. Long-Term Outcomes After Stereotactic Radiosurgery for Spine Metastases: Radiation Dose-Response for Late Toxicity. Int J Radiat Oncol Biol Phys 2018;101(3):602-609. (In eng). DOI: 10.1016/j.ijrobp.2018.02.035.

145. Liss AL, Marsh RB, Kapadia NS, et al. Decreased Lung Perfusion After Breast/Chest Wall Irradiation: Quantitative Results From a Prospective Clinical Trial. Int J Radiat Oncol Biol Phys 2017;97(2):296-302. (In eng). DOI: 10.1016/j.ijrobp.2016.10.012.

146. Liu J, Zhao Q, Deng W, et al. Radiation-related lymphopenia is associated with spleen irradiation dose during radiotherapy in patients with hepatocellular carcinoma. Radiat Oncol 2017;12(1):90. (In eng). DOI: 10.1186/s13014-017-0824-x.

147. Liu Y, Wang W, Shiue K, et al. Risk factors for symptomatic radiation pneumonitis after stereotactic body radiation therapy (SBRT) in patients with non-small cell lung cancer. Radiother Oncol 2021;156:231-238. (In eng). DOI: 10.1016/j.radonc.2020.10.015.

148. Lo Q, Hee L, Batumalai V, et al. Subclinical cardiac dysfunction detected by strain imaging during breast irradiation with persistent changes 6 weeks after treatment. Int J Radiat Oncol Biol Phys 2015;92(2):268-76. (In eng). DOI: 10.1016/j.ijrobp.2014.11.016.

149. Loi M, Magallon-Baro A, Suker M, Van Eijck C, Hoogeman M, Nuyttens JJ. Daily dose to organs at risk predicts acute toxicity in pancreatic stereotactic radiotherapy. Acta Oncol 2020;59(8):944-948. (In eng). DOI: 10.1080/0284186x.2020.1742931.

150. Lucas JT, Jr., Fernandez-Pineda I, Tinkle CL, et al. Late toxicity and outcomes following radiation therapy for chest wall sarcomas in pediatric patients. Pract Radiat Oncol 2017;7(6):411-417. (In eng). DOI: 10.1016/j.prro.2017.04.015.

151. Luna JM, Chao HH, Diffenderfer ES, et al. Predicting radiation pneumonitis in locally advanced stage II-III non-small cell lung cancer using machine learning. Radiother Oncol 2019;133:106-112. (In eng). DOI: 10.1016/j.radonc.2019.01.003.

152. Luo Y, El Naqa I, McShan DL, et al. Unraveling biophysical interactions of radiation pneumonitis in non-small-cell lung cancer via Bayesian network analysis. Radiother Oncol 2017;123(1):85-92. (In eng). DOI: 10.1016/j.radonc.2017.02.004.

153. Ma JT, Sun L, Sun X, et al. Is pulmonary artery a dose-limiting organ at risk in non-small cell lung cancer patients treated with definitive radiotherapy? Radiat Oncol 2017;12(1):34. (In eng). DOI: 10.1186/s13014-017-0772-5.

154. Ma TM, Grimm J, McIntyre R, et al. A prospective evaluation of hippocampal radiation dose volume effects and memory deficits following cranial irradiation. Radiother Oncol 2017;125(2):234-240. (In eng). DOI: 10.1016/j.radonc.2017.09.035.

155. Mak KS, Chen YH, Catalano PJ, et al. Dosimetric Inhomogeneity Predicts for Long-Term Breast Pain After Breast-Conserving Therapy. Int J Radiat Oncol Biol Phys 2015;93(5):1087-95. (In eng). DOI: 10.1016/j.ijrobp.2014.05.021.

156. Manea E, Escande A, Bockel S, et al. Risk of Late Urinary Complications Following Image Guided Adaptive Brachytherapy for Locally Advanced Cervical Cancer: Refining Bladder Dose-Volume Parameters. Int J Radiat Oncol Biol Phys 2018;101(2):411-420. (In eng). DOI: 10.1016/j.ijrobp.2018.02.004.

157. Manyam BV, Verdecchia K, Videtic GMM, et al. Validation of RTOG 0813 Proximal Bronchial Tree Constraints for Pulmonary Toxicity With Stereotactic Body Radiation Therapy for Central Non-small Cell Lung Cancer. Int J Radiat Oncol Biol Phys 2020;107(1):72-78. (In eng). DOI: 10.1016/j.ijrobp.2020.01.009.

158. Manyam BV, Videtic GMM, Verdecchia K, Reddy CA, Woody NM, Stephans KL. Effect of Tumor Location and Dosimetric Predictors for Chest Wall Toxicity in Single-Fraction Stereotactic Body Radiation Therapy for Stage I Non-Small Cell Lung Cancer. Pract Radiat Oncol 2019;9(2):e187-e195. (In eng). DOI: 10.1016/j.prro.2018.11.011.

159. Matuschek C, Bölke E, Geigis C, et al. Influence of dosimetric and clinical criteria on the requirement of artificial nutrition during radiotherapy of head and neck cancer patients. Radiother Oncol 2016;120(1):28-35. (In eng). DOI: 10.1016/j.radonc.2016.05.017.

160. Mazeron R, Fokdal LU, Kirchheiner K, et al. Dose-volume effect relationships for late rectal morbidity in patients treated with chemoradiation and MRI-guided adaptive brachytherapy for locally advanced cervical cancer: Results from the prospective multicenter EMBRACE study. Radiother Oncol 2016;120(3):412-419. (In eng). DOI: 10.1016/j.radonc.2016.06.006.

161. Mazeron R, Gouy S, Chargari C, et al. Post radiation hysterectomy in locally advanced cervical cancer: Outcomes and dosimetric impact. Radiother Oncol 2016;120(3):460-466. (In eng). DOI: 10.1016/j.radonc.2016.07.010.

162. Mazeron R, Maroun P, Castelnau-Marchand P, et al. Pulsed-dose rate image-guided adaptive brachytherapy in cervical cancer: Dose-volume effect relationships for the rectum and bladder. Radiother Oncol 2015;116(2):226-32. (In eng). DOI: 10.1016/j.radonc.2015.06.027.

163. McDonald AM, Baker CB, Popple RA, Cardan RA, Fiveash JB. Increased radiation dose heterogeneity within the prostate predisposes to urethral strictures in patients receiving moderately hypofractionated prostate radiation therapy. Pract Radiat Oncol 2015;5(5):338-342. (In eng). DOI: 10.1016/j.prro.2015.02.010.

164. McDonald MW, Linton OR, Calley CS. Dose-volume relationships associated with temporal lobe radiation necrosis after skull base proton beam therapy. Int J Radiat Oncol Biol Phys 2015;91(2):261-7. (In eng). DOI: 10.1016/j.ijrobp.2014.10.011.

165. McFarlane MR, Hochstedler KA, Laucis AM, et al. Predictors of Pneumonitis After Conventionally Fractionated Radiotherapy for Locally Advanced Lung Cancer. Int J Radiat Oncol Biol Phys 2021;111(5):1176-1185. (In eng). DOI: 10.1016/j.ijrobp.2021.07.1691.

166. McWilliam A, Khalifa J, Vasquez Osorio E, et al. Novel Methodology to Investigate the Effect of Radiation Dose to Heart Substructures on Overall Survival. Int J Radiat Oncol Biol Phys 2020;108(4):1073-1081. (In eng). DOI: 10.1016/j.ijrobp.2020.06.031.

167. Meng Y, Yang H, Wang W, et al. Excluding PTV from lung volume may better predict radiation pneumonitis for intensity modulated radiation therapy in lung cancer patients. Radiat Oncol 2019;14(1):7. (In eng). DOI: 10.1186/s13014-018-1204-x.

168. Mirjolet C, Walker PM, Gauthier M, et al. Absolute volume of the rectum and AUC from rectal DVH between 25Gy and 50Gy predict acute gastrointestinal toxicity with IG-IMRT in prostate cancer. Radiat Oncol 2016;11(1):145. (In eng). DOI: 10.1186/s13014-016-0721-8.

169. Miyasaka Y, Okonogi N, Fukahori M, et al. Pelvic insufficiency fractures following carbon-ion radiotherapy for uterine carcinomas. Radiother Oncol 2021;156:56-61. (In eng). DOI: 10.1016/j.radonc.2020.11.030.

170. Moignier A, Broggio D, Derreumaux S, et al. Coronary stenosis risk analysis following Hodgkin lymphoma radiotherapy: A study based on patient specific artery segments dose calculation. Radiother Oncol 2015;117(3):467-72. (In eng). DOI: 10.1016/j.radonc.2015.07.043.

171. Moulton CR, House MJ, Lye V, et al. Prostate external beam radiotherapy combined with high-dose-rate brachytherapy: dose-volume parameters from deformably-registered plans correlate with late gastrointestinal complications. Radiat Oncol 2016;11(1):144. (In eng). DOI: 10.1186/s13014-016-0719-2.

172. Mouttet-Audouard R, Lacornerie T, Tresch E, et al. What is the normal tissues morbidity following Helical Intensity Modulated Radiation Treatment for cervical cancer? Radiother Oncol 2015;115(3):386-91. (In eng). DOI: 10.1016/j.radonc.2015.02.010.

173. Murofushi KN, Oguchi M, Gosho M, Kozuka T, Sakurai H. Radiation-induced bronchiolitis obliterans organizing pneumonia (BOOP) syndrome in breast cancer patients is associated with age. Radiat Oncol 2015;10:103. (In eng). DOI: 10.1186/s13014-015-0393-9.

174. Musunuru HB, Davidson M, Cheung P, et al. Predictive Parameters of Symptomatic Hematochezia Following 5-Fraction Gantry-Based SABR in Prostate Cancer. Int J Radiat Oncol Biol Phys 2016;94(5):1043-51. (In eng). DOI: 10.1016/j.ijrobp.2015.12.010.

175. Mylona E, Acosta O, Lizee T, et al. Voxel-Based Analysis for Identification of Urethrovesical Subregions Predicting Urinary Toxicity After Prostate Cancer Radiation Therapy. Int J Radiat Oncol Biol Phys 2019;104(2):343-354. (In eng). DOI: 10.1016/j.ijrobp.2019.01.088.

176. Mylona E, Ebert M, Kennedy A, et al. Rectal and Urethro-Vesical Subregions for Toxicity Prediction After Prostate Cancer Radiation Therapy: Validation of Voxel-Based Models in an Independent Population. Int J Radiat Oncol Biol Phys 2020;108(5):1189-1195. (In eng). DOI: 10.1016/j.ijrobp.2020.07.019.

177. Nagore G, Lopez Guerra JL, Krumina E, et al. High dose rate brachytherapy for prostate cancer: A prospective toxicity evaluation of a one day schedule including two 13.5 Gy fractions. Radiother Oncol 2018;127(2):219-224. (In eng). DOI: 10.1016/j.radonc.2018.03.022.

178. Nakajima M, Yamamoto N, Hayashi K, et al. Carbon-ion radiotherapy for non-small cell lung cancer with interstitial lung disease: a retrospective analysis. Radiat Oncol 2017;12(1):144. (In eng). DOI: 10.1186/s13014-017-0881-1.

179. Nakatsugawa M, Cheng Z, Kiess A, et al. The Needs and Benefits of Continuous Model Updates on the Accuracy of RT-Induced Toxicity Prediction Models Within a Learning Health System. Int J Radiat Oncol Biol Phys 2019;103(2):460-467. (In eng). DOI: 10.1016/j.ijrobp.2018.09.038.

180. Nanda RH, Ganju RG, Schreibmann E, et al. Correlation of Acute and Late Brainstem Toxicities With Dose-Volume Data for Pediatric Patients With Posterior Fossa Malignancies. Int J Radiat Oncol Biol Phys 2017;98(2):360-366. (In eng). DOI: 10.1016/j.ijrobp.2017.02.092.

181. NB KJ, Pötter R, Spampinato S, et al. Dose-Volume Effects and Risk Factors for Late Diarrhea in Cervix Cancer Patients After Radiochemotherapy With Image Guided Adaptive Brachytherapy in the EMBRACE I Study. Int J Radiat Oncol Biol Phys 2021;109(3):688-700. (In eng). DOI: 10.1016/j.ijrobp.2020.10.006.

182. Newman NB, Sidhu MK, Baby R, et al. Long-Term Bone Marrow Suppression During Postoperative Chemotherapy in Rectal Cancer Patients After Preoperative Chemoradiation Therapy. Int J Radiat Oncol Biol Phys 2016;94(5):1052-60. (In eng). DOI: 10.1016/j.ijrobp.2015.12.374.

183. Ng LW, Wong KK, Ally Wu CL, Sposto R, Olch AJ. Dose Sculpting Intensity Modulated Radiation Therapy for Vertebral Body Sparing in Children With Neuroblastoma. Int J Radiat Oncol Biol Phys 2018;101(3):550-557. (In eng). DOI: 10.1016/j.ijrobp.2018.02.015.

184. Nguyen SM, Sison J, Jones M, et al. Lens Dose-Response Prediction Modeling and Cataract Incidence in Patients With Retinoblastoma After Lens-Sparing or Whole-Eye Radiation Therapy. Int J Radiat Oncol Biol Phys 2019;103(5):1143-1150. (In eng). DOI: 10.1016/j.ijrobp.2018.12.004.

185. Nieder C, Imingen KS, Mannsåker B, Yobuta R, Haukland E. Risk factors for esophagitis after hypofractionated palliative (chemo) radiotherapy for non-small cell lung cancer. Radiat Oncol 2020;15(1):91. (In eng). DOI: 10.1186/s13014-020-01550-2.

186. Niedzielski JS, Wei X, Xu T, et al. Development and application of an elastic net logistic regression model to investigate the impact of cardiac substructure dose on radiation-induced pericardial effusion in patients with NSCLC. Acta Oncol 2020;59(10):1193-1200. (In eng). DOI: 10.1080/0284186x.2020.1794034.

187. Niedzielski JS, Yang J, Mohan R, et al. Differences in Normal Tissue Response in the Esophagus Between Proton and Photon Radiation Therapy for Non-Small Cell Lung Cancer Using In Vivo Imaging Biomarkers. Int J Radiat Oncol Biol Phys 2017;99(4):1013-1020. (In eng). DOI: 10.1016/j.ijrobp.2017.07.005.

188. Nilsson MP, Johnsson A, Scherman J. Sarcopenia and dosimetric parameters in relation to treatment-related leukopenia and survival in anal cancer. Radiat Oncol 2021;16(1):152. (In eng). DOI: 10.1186/s13014-021-01876-5.

189. Ning MS, Tang L, Gomez DR, et al. Incidence and Predictors of Pericardial Effusion After Chemoradiation Therapy for Locally Advanced Non-Small Cell Lung Cancer. Int J Radiat Oncol Biol Phys 2017;99(1):70-79. (In eng). DOI: 10.1016/j.ijrobp.2017.05.022.

190. O'Reilly S, Jain V, Huang Q, et al. Dose to Highly Functional Ventilation Zones Improves Prediction of Radiation Pneumonitis for Proton and Photon Lung Cancer Radiation Therapy. Int J Radiat Oncol Biol Phys 2020;107(1):79-87. (In eng). DOI: 10.1016/j.ijrobp.2020.01.014.

191. Okonogi N, Fukahori M, Wakatsuki M, et al. Dose constraints in the rectum and bladder following carbon-ion radiotherapy for uterus carcinoma: a retrospective pooled analysis. Radiat Oncol 2018;13(1):119. (In eng). DOI: 10.1186/s13014-018-1061-7.

192. Olsen JR, Moughan J, Myerson R, et al. Predictors of Radiation Therapy-Related Gastrointestinal Toxicity From Anal Cancer Dose-Painted Intensity Modulated Radiation Therapy: Secondary Analysis of NRG Oncology RTOG 0529. Int J Radiat Oncol Biol Phys 2017;98(2):400-408. (In eng). DOI: 10.1016/j.ijrobp.2017.02.005.

193. Ono T, Nakamura T, Yamaguchi H, et al. Clinical results of proton beam therapy for elderly patients with non-small cell lung cancer. Radiat Oncol 2018;13(1):19. (In eng). DOI: 10.1186/s13014-018-0967-4.

194. Osman SO, Horn S, Brady D, et al. Prostate cancer treated with brachytherapy; an exploratory study of dose-dependent biomarkers and quality of life. Radiat Oncol 2017;12(1):53. (In eng). DOI: 10.1186/s13014-017-0792-1.

195. Osmundson EC, Wu Y, Luxton G, Bazan JG, Koong AC, Chang DT. Predictors of toxicity associated with stereotactic body radiation therapy to the central hepatobiliary tract. Int J Radiat Oncol Biol Phys 2015;91(5):986-94. (In eng). DOI: 10.1016/j.ijrobp.2014.11.028.

196. Otani K, Teshima T, Ito Y, et al. Risk factors for vertebral compression fractures in preoperative chemoradiotherapy with gemcitabine for pancreatic cancer. Radiother Oncol 2016;118(3):424-9. (In eng). DOI: 10.1016/j.radonc.2016.01.006.

197. Otter S, Schick U, Gulliford S, et al. Evaluation of the Risk of Grade 3 Oral and Pharyngeal Dysphagia Using Atlas-Based Method and Multivariate Analyses of Individual Patient Dose Distributions. Int J Radiat Oncol Biol Phys 2015;93(3):507-15. (In eng). DOI: 10.1016/j.ijrobp.2015.07.2263.

198. Pan Y, Brink C, Knap M, et al. Acute esophagitis for patients with local-regional advanced non small cell lung cancer treated with concurrent chemoradiotherapy. Radiother Oncol 2016;118(3):465-70. (In eng). DOI: 10.1016/j.radonc.2016.01.007.

199. Pao TH, Chang WL, Chiang NJ, et al. Cardiac radiation dose predicts survival in esophageal squamous cell carcinoma treated by definitive concurrent chemotherapy and intensity modulated radiotherapy. Radiat Oncol 2020;15(1):221. (In eng). DOI: 10.1186/s13014-020-01664-7.

200. Park Y, Kim HJ, Chang AR. Predictors of chest wall toxicity after stereotactic ablative radiotherapy using real-time tumor tracking for lung tumors. Radiat Oncol 2017;12(1):66. (In eng). DOI: 10.1186/s13014-017-0803-2.

201. Parker SM, Siochi RA, Wen S, Mattes MD. Impact of Tumor Size on Local Control and Pneumonitis After Stereotactic Body Radiation Therapy for Lung Tumors. Pract Radiat Oncol 2019;9(1):e90-e97. (In eng). DOI: 10.1016/j.prro.2018.09.003.

202. Patel AK, Ling DC, Richman AH, et al. Hypofractionated Whole-Breast Irradiation in Large-Breasted Women-Is There a Dosimetric Predictor for Acute Skin Toxicities? Int J Radiat Oncol Biol Phys 2019;103(1):71-77. (In eng). DOI: 10.1016/j.ijrobp.2018.08.024.

203. Paul S, Bodner WR, Garg M, Tang J, Ohri N. Cardiac Irradiation Predicts Activity Decline in Patients Receiving Concurrent Chemoradiation for Locally Advanced Lung Cancer. Int J Radiat Oncol Biol Phys 2020;108(3):597-601. (In eng). DOI: 10.1016/j.ijrobp.2020.05.042.

204. Pauli N, Olsson C, Pettersson N, et al. Risk structures for radiation-induced trismus in head and neck cancer. Acta Oncol 2016;55(6):788-92. (In eng). DOI: 10.3109/0284186x.2016.1143564.

205. Paximadis P, Schipper M, Matuszak M, et al. Dosimetric predictors for acute esophagitis during radiation therapy for lung cancer: Results of a large statewide observational study. Pract Radiat Oncol 2018;8(3):167-173. (In eng). DOI: 10.1016/j.prro.2017.07.010.

206. Peng X, Zhou S, Liu S, et al. Dose-volume analysis of predictors for acute anal toxicity after radiotherapy in prostate cancer patients. Radiat Oncol 2019;14(1):174. (In eng). DOI: 10.1186/s13014-019-1374-1.

207. Peters M, van der Voort van Zyp J, Hoekstra C, et al. Urethral and bladder dosimetry of total and focal salvage Iodine-125 prostate brachytherapy: Late toxicity and dose constraints. Radiother Oncol 2015;117(2):262-9. (In eng). DOI: 10.1016/j.radonc.2015.08.018.

208. Pinnix CC, Cella L, Andraos TY, et al. Predictors of Hypothyroidism in Hodgkin Lymphoma Survivors After Intensity Modulated Versus 3-Dimensional Radiation Therapy. Int J Radiat Oncol Biol Phys 2018;101(3):530-540. (In eng). DOI: 10.1016/j.ijrobp.2018.03.003.

209. Pinnix CC, Smith GL, Milgrom S, et al. Predictors of radiation pneumonitis in patients receiving intensity modulated radiation therapy for Hodgkin and non-Hodgkin lymphoma. Int J Radiat Oncol Biol Phys 2015;92(1):175-82. (In eng). DOI: 10.1016/j.ijrobp.2015.02.010.

210. Polishchuk AL, Mishra KK, Weinberg V, et al. Temporal Evolution and Dose-Volume Histogram Predictors of Visual Acuity After Proton Beam Radiation Therapy of Uveal Melanoma. Int J Radiat Oncol Biol Phys 2017;97(1):91-97. (In eng). DOI: 10.1016/j.ijrobp.2016.09.019.

211. Prayongrat A, Kobashi K, Ito YM, et al. The normal tissue complication probability model-based approach considering uncertainties for the selective use of radiation modality in primary liver cancer patients. Radiother Oncol 2019;135:100-106. (In eng). DOI: 10.1016/j.radonc.2019.03.003.

212. Qi XS, Wang JP, Gomez CL, et al. Plan quality and dosimetric association of patient-reported rectal and urinary toxicities for prostate stereotactic body radiotherapy. Radiother Oncol 2016;121(1):113-117. (In eng). DOI: 10.1016/j.radonc.2016.08.012.

213. Rahimi A, Zhang Y, Kim DW, et al. Risk Factors for Fat Necrosis After Stereotactic Partial Breast Irradiation for Early-Stage Breast Cancer in a Phase 1 Clinical Trial. Int J Radiat Oncol Biol Phys 2020;108(3):697-706. (In eng). DOI: 10.1016/j.ijrobp.2020.05.025.

214. Ramlov A, Pedersen EM, Røhl L, et al. Risk Factors for Pelvic Insufficiency Fractures in Locally Advanced Cervical Cancer Following Intensity Modulated Radiation Therapy. Int J Radiat Oncol Biol Phys 2017;97(5):1032-1039. (In eng). DOI: 10.1016/j.ijrobp.2017.01.026.

215. Rao SD, Saleh ZH, Setton J, et al. Dose-volume factors correlating with trismus following chemoradiation for head and neck cancer. Acta Oncol 2016;55(1):99-104. (In eng). DOI: 10.3109/0284186x.2015.1037864.

216. Reis T, Khazzaka E, Welzel G, Wenz F, Hofheinz RD, Mai S. Acute small-bowel toxicity during neoadjuvant combined radiochemotherapy in locally advanced rectal cancer: determination of optimal dose-volume cut-off value predicting grade 2-3 diarrhoea. Radiat Oncol 2015;10:30. (In eng). DOI: 10.1186/s13014-015-0336-5.

217. Repka MC, Kole TP, Lee J, et al. Predictors of acute urinary symptom flare following stereotactic body radiation therapy (SBRT) in the definitive treatment of localized prostate cancer. Acta Oncol 2017;56(8):1136-1138. (In eng). DOI: 10.1080/0284186x.2017.1299221.

218. Ribeiro I, Janssen H, De Brabandere M, et al. Long term experience with 3D image guided brachytherapy and clinical outcome in cervical cancer patients. Radiother Oncol 2016;120(3):447-454. (In eng). DOI: 10.1016/j.radonc.2016.04.016.

219. Rijkmans EC, Marijnen CAM, van Triest B, et al. Predictive factors for response and toxicity after brachytherapy for rectal cancer; results from the HERBERT study. Radiother Oncol 2019;133:176-182. (In eng). DOI: 10.1016/j.radonc.2019.01.034.

220. Robinson M, Muirhead R, Jacobs C, et al. Response of FDG avid pelvic bone marrow to concurrent chemoradiation for anal cancer. Radiother Oncol 2020;143:19-23. (In eng). DOI: 10.1016/j.radonc.2019.08.016.

221. Robinson M, Sabbagh A, Muirhead R, Durrant L, Van den Heuvel F, Hawkins M. Modeling early haematologic adverse events in conformal and intensity-modulated pelvic radiotherapy in anal cancer. Radiother Oncol 2015;117(2):246-51. (In eng). DOI: 10.1016/j.radonc.2015.09.009.

222. Rodríguez-López JL, Ling DC, Keller A, et al. Ureteral stenosis after 3D MRI-based brachytherapy for cervical cancer - Have we identified all the risk factors? Radiother Oncol 2021;155:86-92. (In eng). DOI: 10.1016/j.radonc.2020.10.010.

223. Romano E, Simon R, Minard-Colin V, et al. Analysis of Radiation Dose/Volume Effect Relationship for Anorectal Morbidity in Children Treated for Pelvic Malignancies. Int J Radiat Oncol Biol Phys 2021;109(1):231-241. (In eng). DOI: 10.1016/j.ijrobp.2020.08.033.

224. Rønjom MF, Brink C, Bentzen SM, et al. External validation of a normal tissue complication probability model for radiation-induced hypothyroidism in an independent cohort. Acta Oncol 2015;54(9):1301-9. (In eng). DOI: 10.3109/0284186x.2015.1064160.

225. Rose B, Mitra D, Hong TS, et al. Irradiation of anatomically defined pelvic subsites and acute hematologic toxicity in anal cancer patients undergoing chemoradiation. Pract Radiat Oncol 2017;7(5):e291-e297. (In eng). DOI: 10.1016/j.prro.2017.03.008.

226. Rose BS, Jee KW, Niemierko A, et al. Irradiation of FDG-PET-Defined Active Bone Marrow Subregions and Acute Hematologic Toxicity in Anal Cancer Patients Undergoing Chemoradiation. Int J Radiat Oncol Biol Phys 2016;94(4):747-54. (In eng). DOI: 10.1016/j.ijrobp.2015.12.006.

227. Rosen BS, Hawkins PG, Polan DF, et al. Early Changes in Serial CBCT-Measured Parotid Gland Biomarkers Predict Chronic Xerostomia After Head and Neck Radiation Therapy. Int J Radiat Oncol Biol Phys 2018;102(4):1319-1329. (In eng). DOI: 10.1016/j.ijrobp.2018.06.048.

228. Rossi L, Bijman R, Schillemans W, et al. Texture analysis of 3D dose distributions for predictive modelling of toxicity rates in radiotherapy. Radiother Oncol 2018;129(3):548-553. (In eng). DOI: 10.1016/j.radonc.2018.07.027.

229. Rudra S, Hui C, Rao YJ, et al. Effect of Radiation Treatment Volume Reduction on Lymphopenia in Patients Receiving Chemoradiotherapy for Glioblastoma. Int J Radiat Oncol Biol Phys 2018;101(1):217-225. (In eng). DOI: 10.1016/j.ijrobp.2018.01.069.

230. Rwigema JM, Langendijk JA, Paul van der Laan H, Lukens JN, Swisher-McClure SD, Lin A. A Model-Based Approach to Predict Short-Term Toxicity Benefits With Proton Therapy for Oropharyngeal Cancer. Int J Radiat Oncol Biol Phys 2019;104(3):553-562. (In eng). DOI: 10.1016/j.ijrobp.2018.12.055.

231. Ryckman JM, Baine M, Carmicheal J, et al. Correlation of dosimetric factors with the development of symptomatic radiation pneumonitis in stereotactic body radiotherapy. Radiat Oncol 2020;15(1):33. (In eng). DOI: 10.1186/s13014-020-1479-6.

232. Saha A, Beasley M, Hatton N, et al. Clinical and dosimetric predictors of radiation pneumonitis in early-stage lung cancer treated with Stereotactic Ablative radiotherapy (SABR) - An analysis of UK's largest cohort of lung SABR patients. Radiother Oncol 2021;156:153-159. (In eng). DOI: 10.1016/j.radonc.2020.12.015.

233. Sapir E, Tao Y, Feng F, et al. Predictors of Dysgeusia in Patients With Oropharyngeal Cancer Treated With Chemotherapy and Intensity Modulated Radiation Therapy. Int J Radiat Oncol Biol Phys 2016;96(2):354-361. (In eng). DOI: 10.1016/j.ijrobp.2016.05.011.

234. Schaake W, van der Schaaf A, van Dijk LV, Bongaerts AH, van den Bergh AC, Langendijk JA. Normal tissue complication probability (NTCP) models for late rectal bleeding, stool frequency and fecal incontinence after radiotherapy in prostate cancer patients. Radiother Oncol 2016;119(3):381-7. (In eng). DOI: 10.1016/j.radonc.2016.04.005.

235. Schaub SK, Apisarnthanarax S, Price RG, et al. Functional Liver Imaging and Dosimetry to Predict Hepatotoxicity Risk in Cirrhotic Patients With Primary Liver Cancer. Int J Radiat Oncol Biol Phys 2018;102(4):1339-1348. (In eng). DOI: 10.1016/j.ijrobp.2018.08.029.

236. Schiller K, Specht HM, Haller B, et al. Correlation between delivered radiation doses to the brainstem or vestibular organ and nausea & vomiting toxicity in patients with head and neck cancers - an observational clinical trial. Radiat Oncol 2017;12(1):113. (In eng). DOI: 10.1186/s13014-017-0846-4.

237. Schytte T, Bentzen SM, Brink C, Hansen O. Changes in pulmonary function after definitive radiotherapy for NSCLC. Radiother Oncol 2015;117(1):23-8. (In eng). DOI: 10.1016/j.radonc.2015.09.029.

238. Seibert TM, Karunamuni R, Bartsch H, et al. Radiation Dose-Dependent Hippocampal Atrophy Detected With Longitudinal Volumetric Magnetic Resonance Imaging. Int J Radiat Oncol Biol Phys 2017;97(2):263-269. (In eng). DOI: 10.1016/j.ijrobp.2016.10.035.

239. Seibert TM, Karunamuni R, Kaifi S, et al. Cerebral Cortex Regions Selectively Vulnerable to Radiation Dose-Dependent Atrophy. Int J Radiat Oncol Biol Phys 2017;97(5):910-918. (In eng). DOI: 10.1016/j.ijrobp.2017.01.005.

240. Senova S, Aggad M, Golmard JL, et al. Predictors of Trigeminal Neuropathy After Radiosurgery for Vestibular Schwannomas. Int J Radiat Oncol Biol Phys 2016;95(2):721-8. (In eng). DOI: 10.1016/j.ijrobp.2016.01.012.

241. Shaikh T, Churilla TM, Monpara P, Scott WJ, Cohen SJ, Meyer JE. Risk of radiation pneumonitis in patients receiving taxane-based trimodality therapy for locally advanced esophageal cancer. Pract Radiat Oncol 2016;6(6):388-394. (In eng). DOI: 10.1016/j.prro.2016.02.004.

242. Shaikh T, Wang LS, Egleston B, et al. Dosimetric predictors of hematologic toxicity in patients undergoing concurrent gemcitabine-based chemoradiation for localized pancreatic cancer. Pract Radiat Oncol 2016;6(4):e107-e115. (In eng). DOI: 10.1016/j.prro.2015.11.005.

243. Sharma MB, Jensen K, Urbak SF, et al. A multidimensional cohort study of late toxicity after intensity modulated radiotherapy for sinonasal cancer. Radiother Oncol 2020;151:58-65. (In eng). DOI: 10.1016/j.radonc.2020.07.029.

244. Shepherd AF, Iocolano M, Leeman J, et al. Clinical and Dosimetric Predictors of Radiation Pneumonitis in Patients With Non-Small Cell Lung Cancer Undergoing Postoperative Radiation Therapy. Pract Radiat Oncol 2021;11(1):e52-e62. (In eng). DOI: 10.1016/j.prro.2020.09.014.

245. Shinde A, Li R, Han C, Frankel P, Sampath S. Dosimetric Predictors of Genitourinary Toxicity From a Phase I Trial of Prostate Bed Stereotactic Body Radiation Therapy. Pract Radiat Oncol 2021;11(1):e90-e97. (In eng). DOI: 10.1016/j.prro.2020.06.004.

246. Shinoto M, Shioyama Y, Matsunobu A, et al. Dosimetric analysis of upper gastrointestinal ulcer after carbon-ion radiotherapy for pancreatic cancer. Radiother Oncol 2016;120(1):140-4. (In eng). DOI: 10.1016/j.radonc.2016.04.040.

247. Shirai K, Fukata K, Adachi A, et al. Dose-volume histogram analysis of brainstem necrosis in head and neck tumors treated using carbon-ion radiotherapy. Radiother Oncol 2017;125(1):36-40. (In eng). DOI: 10.1016/j.radonc.2017.08.014.

248. Shrestha S, Bates JE, Liu Q, et al. Radiation therapy related cardiac disease risk in childhood cancer survivors: Updated dosimetry analysis from the Childhood Cancer Survivor Study. Radiother Oncol 2021;163:199-208. (In eng). DOI: 10.1016/j.radonc.2021.08.012.

249. Sini C, Fiorino C, Perna L, et al. Dose-volume effects for pelvic bone marrow in predicting hematological toxicity in prostate cancer radiotherapy with pelvic node irradiation. Radiother Oncol 2016;118(1):79-84. (In eng). DOI: 10.1016/j.radonc.2015.11.020.

250. Sini C, Noris Chiorda B, Gabriele P, et al. Patient-reported intestinal toxicity from whole pelvis intensity-modulated radiotherapy: First quantification of bowel dose-volume effects. Radiother Oncol 2017;124(2):296-301. (In eng). DOI: 10.1016/j.radonc.2017.07.005.

251. Söderström K, Nilsson P, Laurell G, Zackrisson B, Jäghagen EL. Dysphagia - Results from multivariable predictive modelling on aspiration from a subset of the ARTSCAN trial. Radiother Oncol 2017;122(2):192-199. (In eng). DOI: 10.1016/j.radonc.2016.09.001.

252. Sommat K, Hussain A, Ong WS, et al. Clinical and dosimetric predictors of physician and patient reported xerostomia following intensity modulated radiotherapy for nasopharyngeal cancer - A prospective cohort analysis. Radiother Oncol 2019;138:149-157. (In eng). DOI: 10.1016/j.radonc.2019.05.023.

253. Sommat K, Ong WS, Hussain A, et al. Thyroid V40 Predicts Primary Hypothyroidism After Intensity Modulated Radiation Therapy for Nasopharyngeal Carcinoma. Int J Radiat Oncol Biol Phys 2017;98(3):574-580. (In eng). DOI: 10.1016/j.ijrobp.2017.03.007.

254. Son CH, Law E, Oh JH, et al. Dosimetric Predictors of Radiation-Induced Vaginal Stenosis After Pelvic Radiation Therapy for Rectal and Anal Cancer. Int J Radiat Oncol Biol Phys 2015;92(3):548-54. (In eng). DOI: 10.1016/j.ijrobp.2015.02.029.

255. Son CH, Melotek JM, Liao C, et al. Bladder dose-volume parameters are associated with urinary incontinence after postoperative intensity modulated radiation therapy for prostate cancer. Pract Radiat Oncol 2016;6(5):e179-e185. (In eng). DOI: 10.1016/j.prro.2015.12.004.

256. Stam B, Peulen H, Guckenberger M, et al. Dose to heart substructures is associated with non-cancer death after SBRT in stage I-II NSCLC patients. Radiother Oncol 2017;123(3):370-375. (In eng). DOI: 10.1016/j.radonc.2017.04.017.

257. Stam B, van der Bijl E, Peulen H, Rossi MMG, Belderbos JSA, Sonke JJ. Dose-effect analysis of radiation induced rib fractures after thoracic SBRT. Radiother Oncol 2017;123(2):176-181. (In eng). DOI: 10.1016/j.radonc.2017.01.004.

258. Stam B, van der Bijl E, van Diessen J, et al. Heart dose associated with overall survival in locally advanced NSCLC patients treated with hypofractionated chemoradiotherapy. Radiother Oncol 2017;125(1):62-65. (In eng). DOI: 10.1016/j.radonc.2017.09.004.

259. Stecklein SR, Shaitelman SF, Babiera GV, et al. Prospective Comparison of Toxicity and Cosmetic Outcome After Accelerated Partial Breast Irradiation With Conformal External Beam Radiotherapy or Single-Entry Multilumen Intracavitary Brachytherapy. Pract Radiat Oncol 2019;9(1):e4-e13. (In eng). DOI: 10.1016/j.prro.2018.08.003.

260. Stervik L, Pettersson N, Scherman J, et al. Analysis of early respiratory-related mortality after radiation therapy of non-small-cell lung cancer: feasibility of automatic data extraction for dose-response studies. Acta Oncol 2020;59(6):628-635. (In eng). DOI: 10.1080/0284186x.2020.1739331.

261. Stoppel G, Eich HT, Matuschek C, et al. Lung toxicity after radiation in childhood: Results of the International Project on Prospective Analysis of Radiotoxicity in Childhood and Adolescence. Radiother Oncol 2017;125(2):286-292. (In eng). DOI: 10.1016/j.radonc.2017.09.026.

262. Su TS, Luo R, Liang P, Cheng T, Zhou Y, Huang Y. A prospective cohort study of hepatic toxicity after stereotactic body radiation therapy for hepatocellular carcinoma. Radiother Oncol 2018;129(1):136-142. (In eng). DOI: 10.1016/j.radonc.2018.02.031.

263. Suresh K, Owen D, Bazzi L, et al. Using Indocyanine Green Extraction to Predict Liver Function After Stereotactic Body Radiation Therapy for Hepatocellular Carcinoma. Int J Radiat Oncol Biol Phys 2018;100(1):131-137. (In eng). DOI: 10.1016/j.ijrobp.2017.09.032.

264. Susko M, Craciunescu O, Meltsner S, et al. Vaginal Dose Is Associated With Toxicity in Image Guided Tandem Ring or Ovoid-Based Brachytherapy. Int J Radiat Oncol Biol Phys 2016;94(5):1099-105. (In eng). DOI: 10.1016/j.ijrobp.2015.12.360.

265. Swanick CW, Allen PK, Tao R, et al. Incidence and predictors of chest wall toxicity after high-dose radiation therapy in 15 fractions. Pract Radiat Oncol 2017;7(1):63-71. (In eng). DOI: 10.1016/j.prro.2016.05.009.

266. Tanenbaum DG, Buchwald ZS, Jhaveri J, et al. Dosimetric Factors Related to Radiation Necrosis After 5-Fraction Radiosurgery for Patients With Resected Brain Metastases. Pract Radiat Oncol 2020;10(1):36-43. (In eng). DOI: 10.1016/j.prro.2019.09.014.

267. Tang X, Li Y, Tian X, et al. Predicting severe acute radiation pneumonitis in patients with non-small cell lung cancer receiving postoperative radiotherapy: Development and internal validation of a nomogram based on the clinical and dose-volume histogram parameters. Radiother Oncol 2019;132:197-203. (In eng). DOI: 10.1016/j.radonc.2018.10.016.

268. Tekatli H, Duijm M, Oomen-de Hoop E, et al. Normal Tissue Complication Probability Modeling of Pulmonary Toxicity After Stereotactic and Hypofractionated Radiation Therapy for Central Lung Tumors. Int J Radiat Oncol Biol Phys 2018;100(3):738-747. (In eng). DOI: 10.1016/j.ijrobp.2017.11.022.

269. Tekatli H, Tetar SU, Nguyen TK, et al. Optimizing SABR delivery for synchronous multiple lung tumors using volumetric-modulated arc therapy. Acta Oncol 2017;56(4):548-554. (In eng). DOI: 10.1080/0284186x.2017.1295166.

270. Thariat J, Grange JD, Mosci C, et al. Visual Outcomes of Parapapillary Uveal Melanomas Following Proton Beam Therapy. Int J Radiat Oncol Biol Phys 2016;95(1):328-335. (In eng). DOI: 10.1016/j.ijrobp.2015.12.011.

271. Thomas M, Defraene G, Lambrecht M, et al. NTCP model for postoperative complications and one-year mortality after trimodality treatment in oesophageal cancer. Radiother Oncol 2019;141:33-40. (In eng). DOI: 10.1016/j.radonc.2019.09.015.

272. Thompson MR, Dumane VA, Lazarev SA, Zia Y, Rosenzweig KE. Dosimetric Correlates of Pulmonary Toxicity in Patients with Malignant Pleural Mesothelioma Receiving Radiation Therapy to the Intact Lungs. Pract Radiat Oncol 2019;9(3):e331-e337. (In eng). DOI: 10.1016/j.prro.2018.12.008.

273. Thor M, Olsson C, Oh JH, et al. Urinary bladder dose-response relationships for patient-reported genitourinary morbidity domains following prostate cancer radiotherapy. Radiother Oncol 2016;119(1):117-22. (In eng). DOI: 10.1016/j.radonc.2016.01.013.

274. Thor M, Olsson CE, Oh JH, et al. Relationships between dose to the gastro-intestinal tract and patient-reported symptom domains after radiotherapy for localized prostate cancer. Acta Oncol 2015;54(9):1326-34. (In eng). DOI: 10.3109/0284186x.2015.1063779.

275. Tjessem KH, Bosse G, Fosså K, et al. Coronary calcium score in 12-year breast cancer survivors after adjuvant radiotherapy with low to moderate heart exposure - Relationship to cardiac radiation dose and cardiovascular risk factors. Radiother Oncol 2015;114(3):328-34. (In eng). DOI: 10.1016/j.radonc.2015.01.006.

276. Toesca DAS, Osmundson EC, von Eyben R, Shaffer JL, Koong AC, Chang DT. Assessment of hepatic function decline after stereotactic body radiation therapy for primary liver cancer. Pract Radiat Oncol 2017;7(3):173-182. (In eng). DOI: 10.1016/j.prro.2016.10.003.

277. Trifiletti DM, Lee CC, Schlesinger D, Larner JM, Xu Z, Sheehan JP. Leukoencephalopathy After Stereotactic Radiosurgery for Brain Metastases. Int J Radiat Oncol Biol Phys 2015;93(4):870-8. (In eng). DOI: 10.1016/j.ijrobp.2015.07.2280.

278. Trip AK, Sikorska K, van Sandick JW, et al. Radiation-induced dose-dependent changes of the spleen following postoperative chemoradiotherapy for gastric cancer. Radiother Oncol 2015;116(2):239-44. (In eng). DOI: 10.1016/j.radonc.2015.07.036.

279. Trivedi SJ, Choudhary P, Lo Q, et al. Persistent reduction in global longitudinal strain in the longer term after radiation therapy in patients with breast cancer. Radiother Oncol 2019;132:148-154. (In eng). DOI: 10.1016/j.radonc.2018.10.023.

280. Tsai PF, Yang CC, Chuang CC, et al. Hippocampal dosimetry correlates with the change in neurocognitive function after hippocampal sparing during whole brain radiotherapy: a prospective study. Radiat Oncol 2015;10:253. (In eng). DOI: 10.1186/s13014-015-0562-x.

281. Tucker SL, Liu A, Gomez D, et al. Impact of heart and lung dose on early survival in patients with non-small cell lung cancer treated with chemoradiation. Radiother Oncol 2016;119(3):495-500. (In eng). DOI: 10.1016/j.radonc.2016.04.025.

282. Uchida Y, Tsugawa T, Tanaka-Mizuno S, et al. Exclusion of emphysematous lung from dose-volume estimates of risk improves prediction of radiation pneumonitis. Radiat Oncol 2017;12(1):160. (In eng). DOI: 10.1186/s13014-017-0891-z.

283. Valstar MH, de Bakker BS, Steenbakkers R, et al. The tubarial salivary glands: A potential new organ at risk for radiotherapy. Radiother Oncol 2021;154:292-298. (In eng). DOI: 10.1016/j.radonc.2020.09.034.

284. van Aken ESM, van der Laan HP, Bijl HP, et al. Risk of ischaemic cerebrovascular events in head and neck cancer patients is associated with carotid artery radiation dose. Radiother Oncol 2021;157:182-187. (In eng). DOI: 10.1016/j.radonc.2021.01.026.

285. van den Bogaard VAB, Spoor DS, van der Schaaf A, et al. The Importance of Radiation Dose to the Atherosclerotic Plaque in the Left Anterior Descending Coronary Artery for Radiation-Induced Cardiac Toxicity of Breast Cancer Patients? Int J Radiat Oncol Biol Phys 2021;110(5):1350-1359. (In eng). DOI: 10.1016/j.ijrobp.2021.03.004.

286. van den Bogaard VAB, van Luijk P, Hummel YM, et al. Cardiac Function After Radiation Therapy for Breast Cancer. Int J Radiat Oncol Biol Phys 2019;104(2):392-400. (In eng). DOI: 10.1016/j.ijrobp.2019.02.003.

287. van der Sande ME, Hupkens BJP, Berbée M, et al. Impact of radiotherapy on anorectal function in patients with rectal cancer following a watch and wait programme. Radiother Oncol 2019;132:79-84. (In eng). DOI: 10.1016/j.radonc.2018.11.017.

288. Veccia A, Caffo O, Fellin G, et al. Impact of post-implant dosimetric parameters on the quality of life of patients treated with low-dose rate brachytherapy for localised prostate cancer: results of a single-institution study. Radiat Oncol 2015;10:130. (In eng). DOI: 10.1186/s13014-015-0434-4.

289. Velec M, Haddad CR, Craig T, et al. Predictors of Liver Toxicity Following Stereotactic Body Radiation Therapy for Hepatocellular Carcinoma. Int J Radiat Oncol Biol Phys 2017;97(5):939-946. (In eng). DOI: 10.1016/j.ijrobp.2017.01.221.

290. Verma V, Bhirud AR, Denniston KA, Bennion NR, Lin C. Quantification of renal function following stereotactic body radiotherapy for pancreatic cancer: secondary dosimetric analysis of a prospective clinical trial. Radiat Oncol 2017;12(1):71. (In eng). DOI: 10.1186/s13014-017-0798-8.

291. Verma V, Lazenby AJ, Zheng D, et al. Dosimetric parameters correlate with duodenal histopathologic damage after stereotactic body radiotherapy for pancreatic cancer: Secondary analysis of a prospective clinical trial. Radiother Oncol 2017;122(3):464-469. (In eng). DOI: 10.1016/j.radonc.2016.12.030.

292. Vivekanandan S, Landau DB, Counsell N, et al. The Impact of Cardiac Radiation Dosimetry on Survival After Radiation Therapy for Non-Small Cell Lung Cancer. Int J Radiat Oncol Biol Phys 2017;99(1):51-60. (In eng). DOI: 10.1016/j.ijrobp.2017.04.026.

293. Walker V, Lairez O, Fondard O, et al. Early detection of subclinical left ventricular dysfunction after breast cancer radiation therapy using speckle-tracking echocardiography: association between cardiac exposure and longitudinal strain reduction (BACCARAT study). Radiat Oncol 2019;14(1):204. (In eng). DOI: 10.1186/s13014-019-1408-8.

294. Walraven I, van den Heuvel M, van Diessen J, et al. Long-term follow-up of patients with locally advanced non-small cell lung cancer receiving concurrent hypofractionated chemoradiotherapy with or without cetuximab. Radiother Oncol 2016;118(3):442-6. (In eng). DOI: 10.1016/j.radonc.2016.02.011.

295. Wan J, Liu K, Li K, Li G, Zhang Z. Can dosimetric parameters predict acute hematologic toxicity in rectal cancer patients treated with intensity-modulated pelvic radiotherapy? Radiat Oncol 2015;10:162. (In eng). DOI: 10.1186/s13014-015-0454-0.

296. Wang J, Miao Y, Ou X, et al. Development and validation of a model for temporal lobe necrosis for nasopharyngeal carcinoma patients with intensity modulated radiation therapy. Radiat Oncol 2019;14(1):42. (In eng). DOI: 10.1186/s13014-019-1250-z.

297. Wang K, Chen RC, Kane BL, et al. Patient and Dosimetric Predictors of Genitourinary and Bowel Quality of Life After Prostate SBRT: Secondary Analysis of a Multi-institutional Trial. Int J Radiat Oncol Biol Phys 2018;102(5):1430-1437. (In eng). DOI: 10.1016/j.ijrobp.2018.07.191.

298. Wang K, Pearlstein KA, Patchett ND, et al. Heart dosimetric analysis of three types of cardiac toxicity in patients treated on dose-escalation trials for Stage III non-small-cell lung cancer. Radiother Oncol 2017;125(2):293-300. (In eng). DOI: 10.1016/j.radonc.2017.10.001.

299. Wang K, Tobillo R, Mavroidis P, et al. Prospective Assessment of Patient-Reported Dry Eye Syndrome After Whole Brain Radiation. Int J Radiat Oncol Biol Phys 2019;105(4):765-772. (In eng). DOI: 10.1016/j.ijrobp.2019.07.015.

300. Wang L, Liang S, Li C, et al. A Novel Nomogram and Risk Classification System Predicting Radiation Pneumonitis in Patients With Esophageal Cancer Receiving Radiation Therapy. Int J Radiat Oncol Biol Phys 2019;105(5):1074-1085. (In eng). DOI: 10.1016/j.ijrobp.2019.08.024.

301. Wang S, Campbell J, Stenmark MH, et al. A model combining age, equivalent uniform dose and IL-8 may predict radiation esophagitis in patients with non-small cell lung cancer. Radiother Oncol 2018;126(3):506-510. (In eng). DOI: 10.1016/j.radonc.2017.12.026.

302. Wang W, Matuszak MM, Hu C, et al. Central Airway Toxicity After High Dose Radiation: A Combined Analysis of Prospective Clinical Trials for Non-Small Cell Lung Cancer. Int J Radiat Oncol Biol Phys 2020;108(3):587-596. (In eng). DOI: 10.1016/j.ijrobp.2020.05.026.

303. Wen DW, Lin L, Mao YP, et al. Normal tissue complication probability (NTCP) models for predicting temporal lobe injury after intensity-modulated radiotherapy in nasopharyngeal carcinoma: A large registry-based retrospective study from China. Radiother Oncol 2021;157:99-105. (In eng). DOI: 10.1016/j.radonc.2021.01.008.

304. Wijsman R, Dankers F, Troost EG, et al. Multivariable normal-tissue complication modeling of acute esophageal toxicity in advanced stage non-small cell lung cancer patients treated with intensity-modulated (chemo-)radiotherapy. Radiother Oncol 2015;117(1):49-54. (In eng). DOI: 10.1016/j.radonc.2015.08.010.

305. Wijsman R, Dankers F, Troost EGC, et al. Inclusion of Incidental Radiation Dose to the Cardiac Atria and Ventricles Does Not Improve the Prediction of Radiation Pneumonitis in Advanced-Stage Non-Small Cell Lung Cancer Patients Treated With Intensity Modulated Radiation Therapy. Int J Radiat Oncol Biol Phys 2017;99(2):434-441. (In eng). DOI: 10.1016/j.ijrobp.2017.04.011.

306. Wolfe AR, Siedow M, Nalin A, et al. Increasing neutrophil-to-lymphocyte ratio following radiation is a poor prognostic factor and directly correlates with splenic radiation dose in pancreatic cancer. Radiother Oncol 2021;158:207-214. (In eng). DOI: 10.1016/j.radonc.2021.02.035.

307. Wu L, Chung YL. Tumor-Infiltrating T Cell Receptor-Beta Repertoires are Linked to the Risk of Late Chemoradiation-Induced Temporal Lobe Necrosis in Locally Advanced Nasopharyngeal Carcinoma. Int J Radiat Oncol Biol Phys 2019;104(1):165-176. (In eng). DOI: 10.1016/j.ijrobp.2019.01.002.

308. Xiao L, Yang G, Chen J, et al. Comparison of predictive powers of functional and anatomic dosimetric parameters for radiation-induced lung toxicity in locally advanced non-small cell lung cancer. Radiother Oncol 2018;129(2):242-248. (In eng). DOI: 10.1016/j.radonc.2018.09.005.

309. Xu B, Guo Y, Chen Y, et al. Is the irradiated small bowel volume still a predictor for acute lower gastrointestinal toxicity during preoperative concurrent chemo-radiotherapy for rectal cancer when using intensity-modulated radiation therapy? Radiat Oncol 2015;10:257. (In eng). DOI: 10.1186/s13014-015-0566-6.

310. Xu YG, Qi SN, Wang SL, et al. Dosimetric and Clinical Outcomes With Intensity Modulated Radiation Therapy After Chemotherapy for Patients With Early-Stage Diffuse Large B-cell Lymphoma of Waldeyer Ring. Int J Radiat Oncol Biol Phys 2016;96(2):379-386. (In eng). DOI: 10.1016/j.ijrobp.2016.05.023.

311. Xue J, Han C, Jackson A, et al. Doses of radiation to the pericardium, instead of heart, are significant for survival in patients with non-small cell lung cancer. Radiother Oncol 2019;133:213-219. (In eng). DOI: 10.1016/j.radonc.2018.10.029.

312. Yamamoto T, Kadoya N, Takeda K, et al. Renal atrophy after stereotactic body radiotherapy for renal cell carcinoma. Radiat Oncol 2016;11:72. (In eng). DOI: 10.1186/s13014-016-0651-5.

313. Yan JJ, Guo SS, Lin DF, et al. Development and validation of a normal tissue complication probability model for acquired nasal cavity stenosis and atresia after radical radiotherapy for nasopharyngeal carcinoma. Radiother Oncol 2021;160:9-17. (In eng). DOI: 10.1016/j.radonc.2021.03.040.

314. Yan K, Ramirez E, Xie XJ, Gu X, Xi Y, Albuquerque K. Predicting severe hematologic toxicity from extended-field chemoradiation of para-aortic nodal metastases from cervical cancer. Pract Radiat Oncol 2018;8(1):13-19. (In eng). DOI: 10.1016/j.prro.2017.07.001.

315. Yaney A, Ayan AS, Pan X, et al. Dosimetric parameters associated with radiation-induced esophagitis in breast cancer patients undergoing regional nodal irradiation. Radiother Oncol 2021;155:167-173. (In eng). DOI: 10.1016/j.radonc.2020.10.042.

316. Yao CY, Zhou GR, Wang LJ, et al. A retrospective dosimetry study of intensity-modulated radiotherapy for nasopharyngeal carcinoma: radiation-induced brainstem injury and dose-volume analysis. Radiat Oncol 2018;13(1):194. (In eng). DOI: 10.1186/s13014-018-1105-z.

317. Yeoh EK, Krol R, Dhillon VS, et al. Predictors of radiation-induced gastrointestinal morbidity: A prospective, longitudinal study following radiotherapy for carcinoma of the prostate. Acta Oncol 2016;55(5):604-10. (In eng). DOI: 10.3109/0284186x.2015.1118658.

318. Yeung R, Bowen SR, Chapman TR, MacLennan GT, Apisarnthanarax S. Chest wall toxicity after hypofractionated proton beam therapy for liver malignancies. Pract Radiat Oncol 2018;8(4):287-293. (In eng). DOI: 10.1016/j.prro.2017.12.007.

319. Yin L, Lu S, Zhu J, Zhang W, Ke G. Ovarian transposition before radiotherapy in cervical cancer patients: functional outcome and the adequate dose constraint. Radiat Oncol 2019;14(1):100. (In eng). DOI: 10.1186/s13014-019-1312-2.

320. Yorke ED, Jackson A, Kuo LC, et al. Heart Dosimetry is Correlated With Risk of Radiation Pneumonitis After Lung-Sparing Hemithoracic Pleural Intensity Modulated Radiation Therapy for Malignant Pleural Mesothelioma. Int J Radiat Oncol Biol Phys 2017;99(1):61-69. (In eng). DOI: 10.1016/j.ijrobp.2017.04.025.

321. Youssef B, Shank J, Reddy JP, et al. Incidence and predictors of Lhermitte's sign among patients receiving mediastinal radiation for lymphoma. Radiat Oncol 2015;10:206. (In eng). DOI: 10.1186/s13014-015-0504-7.

322. Zehentmayr F, Söhn M, Exeli AK, et al. Normal tissue complication models for clinically relevant acute esophagitis (≥ grade 2) in patients treated with dose differentiated accelerated radiotherapy (DART-bid). Radiat Oncol 2015;10:121. (In eng). DOI: 10.1186/s13014-015-0429-1.

323. Zhang L, Johnson J, Gottschalk AR, et al. Receiver operating curves and dose-volume analysis of late toxicity with stereotactic body radiation therapy for prostate cancer. Pract Radiat Oncol 2017;7(2):e109-e116. (In eng). DOI: 10.1016/j.prro.2016.07.004.

324. Zhang YY, Huo WL, Goldberg SI, et al. Brain-Specific Relative Biological Effectiveness of Protons Based on Long-term Outcome of Patients With Nasopharyngeal Carcinoma. Int J Radiat Oncol Biol Phys 2021;110(4):984-992. (In eng). DOI: 10.1016/j.ijrobp.2021.02.018.

325. Zhou L, Chen J, Shen W, et al. Thyroid V(50) is a risk factor for hypothyroidism in patients with nasopharyngeal carcinoma treated with intensity-modulated radiation therapy: a retrospective study. Radiat Oncol 2020;15(1):68. (In eng). DOI: 10.1186/s13014-020-01490-x.

**^195-325^**
